# Metallofullerene photoswitches driven by photoinduced fullerene-to-metal electron transfer†

**DOI:** 10.1039/d0sc07045a

**Published:** 2021-04-30

**Authors:** Michal Zalibera, Frank Ziegs, Sandra Schiemenz, Vasilii Dubrovin, Wolfgang Lubitz, Anton Savitsky, Shihu H. M. Deng, Xue-Bin Wang, Stanislav M. Avdoshenko, Alexey A. Popov

**Affiliations:** Institute of Physical Chemistry and Chemical Physics, Slovak University of Technology in Bratislava Radlinského 9 81237 Bratislava Slovakia michal.zalibera@stuba.sk; Max Planck Institute for Chemical Energy Conversion Mülheim (Ruhr) Germany; Leibniz Institute for Solid State and Materials Research Helmholtzstraße 20 01069 Dresden Germany a.popov@ifw-dresden.de; Faculty of Physics, Technical University Dortmund Otto-Hahn-Str. 4a 44227 Dortmund Germany; Physical Sciences Division, Pacific Northwest National Laboratory Richland Washington 99352 USA

## Abstract

We report on the discovery and detailed exploration of the unconventional photo-switching mechanism in metallofullerenes, in which the energy of the photon absorbed by the carbon cage π-system is transformed to mechanical motion of the endohedral cluster accompanied by accumulation of spin density on the metal atoms. Comprehensive photophysical and electron paramagnetic resonance (EPR) studies augmented by theoretical modelling are performed to address the phenomenon of the light-induced photo-switching and triplet state spin dynamics in a series of Y_*x*_Sc_3−*x*_N@C_80_ (*x* = 0–3) nitride clusterfullerenes. Variable temperature and time-resolved photoluminescence studies revealed a strong dependence of their photophysical properties on the number of Sc atoms in the cluster. All molecules in the series exhibit temperature-dependent luminescence assigned to the near-infrared thermally-activated delayed fluorescence (TADF) and phosphorescence. The emission wavelengths and Stokes shift increase systematically with the number of Sc atoms in the endohedral cluster, whereas the triplet state lifetime and S_1_–T_1_ gap decrease in this row. For Sc_3_N@C_80_, we also applied photoelectron spectroscopy to obtain the triplet state energy as well as the electron affinity. Spin distribution and dynamics in the triplet states are then studied by light-induced pulsed EPR and ENDOR spectroscopies. The spin–lattice relaxation times and triplet state lifetimes are determined from the temporal evolution of the electron spin echo after the laser pulse. Well resolved ENDOR spectra of triplets with a rich structure caused by the hyperfine and quadrupolar interactions with ^14^N, ^45^Sc, and ^89^Y nuclear spins are obtained. The systematic increase of the metal contribution to the triplet spin density from Y_3_N to Sc_3_N found in the ENDOR study points to a substantial fullerene-to-metal charge transfer in the excited state. These experimental results are rationalized with the help of ground-state and time-dependent DFT calculations, which revealed a substantial variation of the endohedral cluster position in the photoexcited states driven by the predisposition of Sc atoms to maximize their spin population.

## Abstract 2

Photoexcitation mechanism of Y_*x*_Sc_3−*x*_N@C_80_ metallofullerenes is studied by variable-temperature photoluminescence, advanced EPR techniques, and DFT calculations, revealing photoinduced rotation of the endohedral cluster.

## Graphical abstract

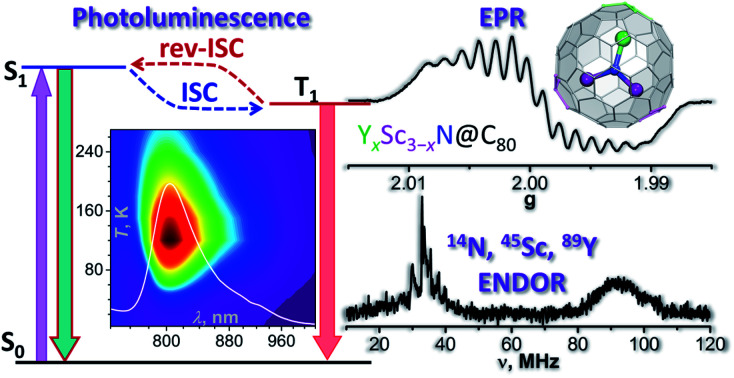

## Introduction

The molecular structure of endohedral metallofullerenes (EMFs) is built from two components, the carbon cage and the endohedral cluster.^1–7^ EMF molecules often have certain structural flexibility associated with the motion of the endohedral species, whose dynamics is apparently decoupled from that of the fullerene cage. For instance, nitride clusterfullerenes M_3_N@C_80_ (M = Sc, Y, lanthanides) exhibit a quasi-free rotation of the M_3_N cluster inside the fullerene near room temperature as evidenced experimentally by NMR spectroscopy^8,9^ and variable-temperature single-crystal X-ray diffraction studies,^10,11^ or computationally by molecular dynamics simulations.^12–15^ But such a dynamic situation does not mean that there is no interaction between the fullerene host and its guest. Endohedral metal atoms transfer their valence electrons to the carbon cage and exhibit substantial d–π orbital overlap and bonding interaction with the fullerene π-system. If the fullerene cage is rather uniform – as is the case for the icosahedral C_80_(*I*_h_) – it has many equivalent or almost equivalent binding sites, and the quasi-free rotation of the cluster means that metal atoms can move easily between these sites. Depending on the temperature and the depths of the energy minima, metal dynamics in EMFs can show different regimes. For M_3_N@C_80_, experimental and computational estimations give the barrier to the cluster rotation on the order of 10 kJ mol^−1^ or less,^9,16,17^ which can be easily reached by the thermal energy in a broad temperature range leading to spontaneous motion of the endohedral clusters down 30–50 K.^18,19^ But the switching of the M_3_N cluster position inside the fullerene can be also induced by more specific external stimuli, which however require experiments to be performed at low temperature to disentangle their effect from thermal motion. Petek *et al.* demonstrated switching of the Sc_3_N cluster positions under the influence of the tunnelling current in the STM study of Sc_3_N@C_80_.^17,20^ Greber *et al.* showed that a magnetic field can affect the orientation of endohedral clusters in HoLu_2_N@C_80_ and TbSc_2_N@C_80_.^18^ Morton *et al.* found that laser irradiation of ErSc_2_N@C_80_ at 5 K changes the ratio of two kinds of EMF species with different EPR spectra.^19^

**Fig. 1:**
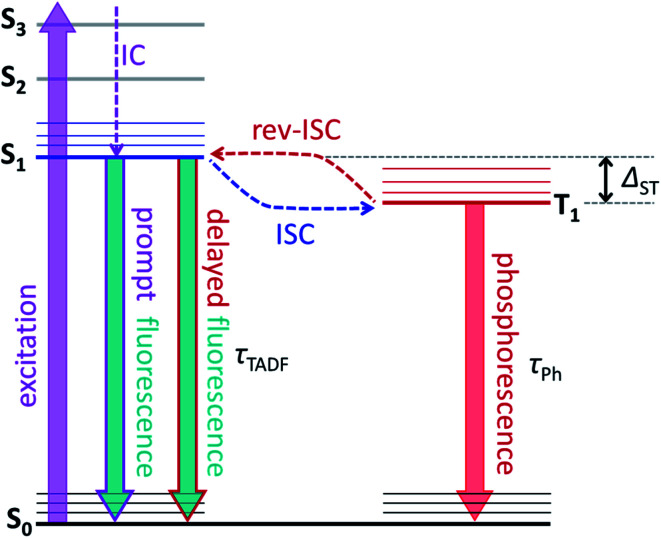
Schematic presentation of the luminescence mechanism with delayed fluorescence. After excitation from the ground singlet state S_0_ to some higher excited singlet state, the system undergoes internal conversion to the lowest-energy excited singlet state S_1_. Then, S_1_ can either decay radiatively to S_0_*via* prompt fluorescence or undergo non-radiative intersystem crossing (ISC) to the triplet state T_1_. Usually, the triplet state decays to S_0_ non-radiatively (this is the most efficient pathway near room temperature) or radiatively, *via* phosphorescence. But some systems can then return from T_1_ back to the S_1_ state in the process known as reverse intersystem crossing (rev-ISC). Radiative decay of S_1_ after rev-ISC gives delayed fluorescence. When the S_1_–T_1_ energy gap (*Δ*_ST_) is sufficiently small, rev-ISC can proceed *via* thermal activation in a suitable temperature range, resulting eventually in thermally-activated delayed fluorescence, TADF. In metallofullerenes studied in this work, prompt fluorescence is not observed, and the luminescence is either TADF alone (at higher temperatures), combination of TADF and phosphorescence (in intermediate temperature range), or phosphorescence alone (below threshold temperature *T*_thr_). In all these processes, the luminescence lifetime corresponds *de facto* to the lifetime of the T_1_ state.

A possibility to switch the position of endohedral units in EMFs with light is very attractive and can potentially lead to control over molecular structure and properties at the level realized in well-established photoswitching materials and molecular machines.^21–26^ But currently, surprisingly little is known about the photophysics of EMFs. Their role as electron donors or acceptors in molecular dyads undergoing photoinduced charge transfer had been studied comprehensively,^27–38^ but the properties of pristine EMFs, including those of iconic Sc_3_N@C_80_, are not well known.^35,37,39–41^ For empty fullerenes, very efficient S_1_ → T_1_ intersystem crossing leads to almost 100% yield of a triplet state after photoexcitation.^42^ The heavy-metal effect in metallofullerenes is expected to further enhance the triplet state formation and facilitate the non-radiative decay. Thus, it is hard to expect that EMFs can show appreciable photoluminescence. The situation can be changed if endohedral metal atoms have low-energy emitting states, and near-infrared lanthanide-based luminescence had been observed in Er- and Tm-based EMFs.^19,43–48^ Surprisingly, Y_3_N@C_80_ was found to show unusually strong luminescence and long fluorescence lifetimes, but the reasons for this unexpected behaviour first remained unclear.^49,50^ Recently we found that the strong photoemission of Y_3_N@C_80_ is caused by the small singlet-triplet S_1_–T_1_ gap of less than 0.1 eV, which enables thermally activated delayed fluorescence (TADF) *via* thermal population of the emitting S_1_ state from the T_1_ “reservoir”.^51^ As the triplet state plays a crucial role in the process, in the same work we also characterized its dynamics and spin distribution by light-induced EPR and ENDOR spectroscopy and found that yttrium atoms have negligible spin populations. TADF is a crucial phenomenon for this work, and Fig. 1 illustrates key processes involved in delayed fluorescence. Note that TADF is a rather common mechanism in many compounds with small S_1_–T_1_ energy difference, including empty fullerenes,^52–56^ and is actively pursued as the way to increase the efficiency of exciton harvesting in OLEDs.^57–59^

In this work, we report on the photophysical and light-induced EPR studies of a series of Y_*x*_Sc_3−*x*_N@C_80_ (*x* = 0–3) nitride clusterfullerenes.^60,61^ Unexpectedly, we found that when Sc atoms are present in the endohedral cluster, the EMF molecules undergo photoswitching. In the photoexcited state, the endohedral cluster acquires a position which ensures the maximal degree of spin density localization on the Sc atoms, and this position is different from the cluster position in the ground-state. We first describe variable-temperatures steady-state and time-resolved photophysical studies, revealing that TADF is a universal scenario for all M_3_N@C_80_ molecules. Then, computations of molecular and electronic structures of EMFs molecules in the ground and excited state are presented, revealing conformer switching and spin density accumulation on the Sc atoms in the excited state. The triplet state energy and close correspondence to the triplet excited state to the Sc_3_N@C_80_^−^ anion are established by photoelectron spectroscopy. Finally, a light-induced time-resolved EPR study addressing dynamics of the photoexcited triplet state is described, concluded by the analysis of the spin distribution in the triplet states using light-induced ENDOR spectroscopy.

## Results and discussion

### Room temperature spectroscopic properties

**Fig. 2:**
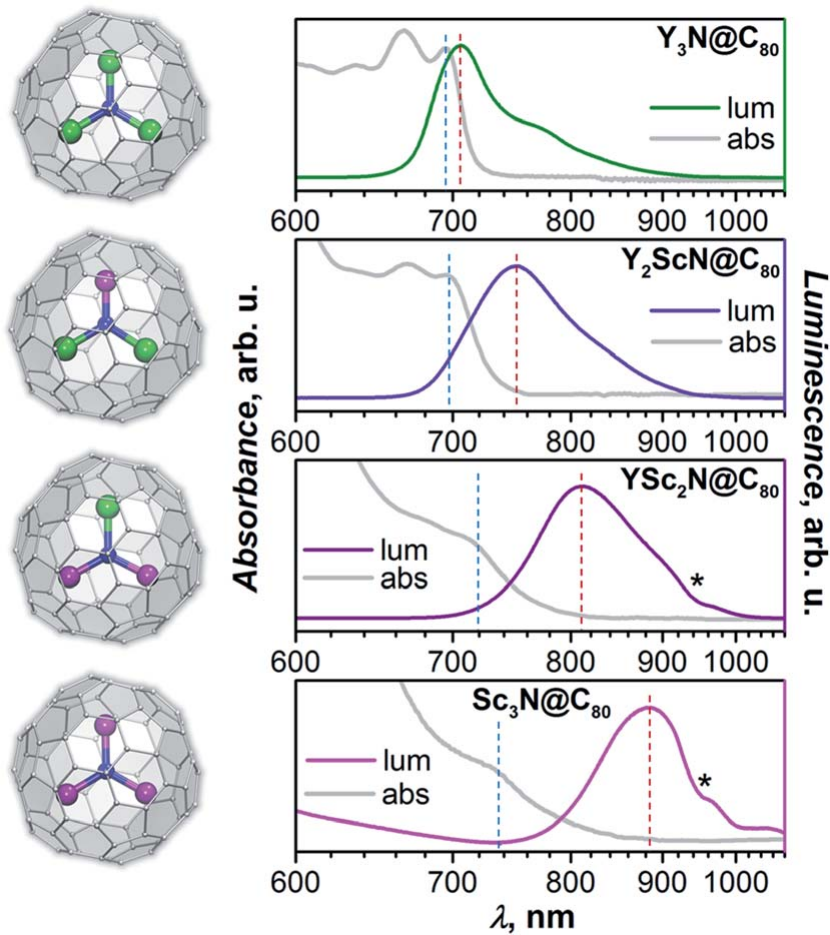
Molecular structures of Y_*x*_Sc_3−*x*_N@C_80_ (*x* = 0–3; Y – green, Sc – magenta, N – blue, C – light grey) and their luminescence (lum) and low-energy absorption (abs) spectra measured in toluene solution at room temperature (laser excitation with *λ*_ex_ = 405 nm). Positions of the lowest-energy absorption features and maximum emission peaks used in calculations of Stokes shifts are marked by blue and red dashed vertical lines, respectively. Asterisks mark an instrumental artefact appearing as a negative peak near 950 nm.

Absorption and photoluminescence (PL) emission spectra of the whole Y_*x*_Sc_3−*x*_N@C_80_ series (*x* = 0–3) are shown in Fig. 2. Despite the same formal charge state of metal and fullerene cage, considerable variations of the spectroscopic properties are observed with the increase of the Sc content in the endohedral nitride cluster as summarized in Table 1 and Fig. 2.

**Table 1:** Selected spectroscopic characteristic of Y_*x*_Sc_3−*x*_N@C_80_ molecules^a^^,^^b^

	Y_3_N@C_80_	Y_2_ScN@C_80_	YSc_2_N@C_80_	Sc_3_N@C_80_
***h***_**8**_ **-Toluene, RT**				
*λ*_abs_, nm (eV)	695 (1.784)	696 (1.782)	713 (1.739)	730 (1.699)
*λ*_lum_, nm (eV)	704 (1.761)	750 (1.653)	810 (1.531)	910 (1.394)
*Δ*_Stokes_, nm (eV)	9 (0.023)	54 (0.128)	97 (0.208)	180 (0.306)

**Polystyrene, VT**				
*λ*_lum_, RT; nm (eV)	705 (1.759)	748 (1.658)	799 (1.552)	869 (1.427)
*λ*_lum_, *T*_max_; nm (eV)	701 (1.769)	752 (1.649)	804 (1.542)	904 (1.372)
*λ*_lum_, 4 K; nm (eV)	739 (1.678)	775 (1.600)	821 (1.510)	915 (1.355)
*Δ*_ST_, optical; eV	0.091 ± 0.006	0.049 ± 0.006	0.032 ± 0.006	n/a
*Δ*_ST_, *τ*-fit; eV	0.098 ± 0.003	0.054 ± 0.002	0.028 ± 0.002	n/a
*τ*_TADF_, RT; μs	1.1	10.5	1.2	0.3
*τ*_Ph_, 4 K; ms	192	78	26	0.4/1.8^c^
*T*_thr_, K	60	40	30	

***d***_**8**_ **-Toluene, 20 K (W-EPR)**				
*τ*_T_1__ ≈ *τ*_Ph_, ms	240 ± 3 (*h*_8_: 180 ± 7)^d^	120 ± 6	38 ± 6	10 ± 6
*τ*_1_, μs	97 ± 1	556 ± 72	1200 ± 140	78 ± 4

a *λ*_abs_ and *λ*_lum_ denote maxima positions of the absorption and luminescence peaks, *Δ*_Stokes_ is the Stokes shift determined from *λ*_abs_ and *λ*_lum_, *T*_max_ is the temperature, at which luminescence has the maximum intensity and is equal to 120 K for Y_3_N@C_80_, Y_2_ScN@C_80_, YSc_2_N@C_80_, and 60 K for Sc_3_N@C_80_; *Δ*_ST_ is the S_1_–T_1_ energy difference determined either form the fluorescence and phosphorescence peak positions in the spectra (“optical”) or by fitting the temperature dependence of luminescence lifetimes with eqn (1) (“*τ*-fit”); *τ*_TADF_ is the fluorescence lifetime at RT; *τ*_Ph_ is the phosphorescence lifetime; *T*_thr_ is the temperature, at which luminescence lifetime reaches a plateau and TADF is quenched; *τ*_1_ is the spin–lattice relaxation time of the triplet state at 20 K in *d*_8_-toluene and *τ*_T_1__ is the triplet state lifetime from the time-resolved light-induced W-band EPR study.

b Precision of the peak positions is ≈±1 nm for absorption in the whole range, ±2 nm for emission at *λ* < 800 nm, ±5 nm for emission at 800 nm < *λ* < 900 nm, and ±10 nm for emission at *λ* > 900 nm; PL lifetime uncertainty is ≈2–3% for single-exponential decays; temperature uncertainty in PL measurements is near 1 K above 100 K, but the measured values can be underestimated by up to 5–8 K close to liquid helium temperature.

c For Sc_3_N@C_80_, the TADF is most probably not completely quenched even at the lowest accessible temperature of 4 K, so the lifetimes may not be the intrinsic *τ*_Ph_.

d Measurement in *h*_8_-toluene gave a *τ*_T_1__ lifetime of 180 ± 7 ms.

Y_3_N@C_80_ shows a well-structured group of absorption features at 600–700 nm with the lowest energy one at 695 nm. The emission spectrum is also relatively narrow with the lowest energy maximum at 704 nm, very close to the absorption maximum. The absorption spectrum of Y_2_ScN@C_80_ is very similar to that of Y_3_N@C_80_ with marginal shifts and somewhat redistributed linewidth. The lowest-energy absorption feature is found at 696 nm. The PL spectrum of Y_2_ScN@C_80_ shows more pronounced differences from that of Y_3_N@C_80_, the peak at 750 nm and a shoulder at 718 nm are considerably broader and shifted to longer wavelengths. Further replacement of Y by Sc results in stronger changes in the absorption spectrum of YSc_2_N@C_80_. The low-energy bands become broader and hence almost smeared. The lowest energy feature can be distinguished at 713 nm. The emission spectrum of YSc_2_N@C_80_ has one broad peak with a maximum at 810 nm. Thus, there is a further red shift of the bands and further broadening. The absorption spectrum of Sc_3_N@C_80_ resembles that of YSc_2_N@C_80_ but is shifted to lower energy, with the single resolved feature at 730 nm. The PL spectrum is further broadened and red-shifted, the maximum is detected near 890 nm (note that due to the strong decrease of the detector sensitivity beyond 800 nm, the position of the maximum for Sc_3_N@C_80_ is less accurate than for other compounds).

A similar variation of absorption spectra with broadening and a red shift was already observed before in the M_*x*_Sc_3−*x*_N@C_80_ series (M = Y, Gd, Dy, Ho, Er, Lu; *x* = 0–3),^60,62–64^ but emission spectra of mixed-metal nitride clusterfullerenes have not been studied yet and reveal a surprisingly strong dependence on the Sc content in the nitride cluster. There is an overall tendency of the red shift for both absorption and emission bands, but the effect for the PL is much stronger (Table 1). As a result, the Stokes shift is also increasing dramatically from 0.02 eV in Y_3_N@C_80_ through 0.13 eV in Y_2_ScN@C_80_ and 0.21 eV in YSc_2_N@C_80_ to ≈0.31 eV in Sc_3_N@C_80_. The reasons for these changes will be analysed and explained below.

Luminescence lifetimes measurements were performed in degassed polystyrene films prepared by mixing fullerene and polymer solutions in CS_2_ and evaporating the solvent under vacuum. For Y_3_N@C_80_, we also performed PL lifetime measurements in degassed toluene solution and obtained the lifetimes similar to those measured in the polymer film (Table S1†). At room temperature, PL lifetimes span the range from 0.3 μs in Sc_3_N@C_80_ to 10.5 μs in Y_2_ScN@C_80_. These values are orders of magnitude longer than normally observed for prompt fluorescence and indicate that the triplet states are likely to be involved in the emission. Thermally-activated delayed fluorescence (TADF) described for Y_3_N@C_80_ (ref. 51) and empty fullerenes^56^ probably take place in all Y_*x*_Sc_3−*x*_N@C_80_ compounds. This hypothesis is further corroborated *via* temperature-dependent PL study.

### Variable temperature photoluminescence (VT-PL)

A small energy difference between T_1_ and S_1_ states enables thermally-activated reversible intersystem crossing from T_1_ to S_1_ with subsequent delayed fluorescence known as TADF (Fig. 1). Unlike the short prompt fluorescence, TADF is characterized by a strongly increased lifetime, which essentially reflects the lifetime of the T_1_ state. In the presence of TADF and the absence of prompt fluorescence, three distinct PL regimes can be expected at different temperatures:

(i) While the temperature is high enough to allow fast thermally-activated rev-ISC from the T_1_ to the S_1_ state, TADF dominates in the PL spectrum. The measured fluorescence lifetime (*τ*_TADF_) in this regime grows with cooling following the increase of the triplet state lifetime at lower temperatures. With the growth of the T_1_ lifetime, the probability that T_1_ decays *via* TADF increases, which leads to the increase of the TADF quantum yield and growth of the PL intensity.

(ii) Once the temperature decreases below a certain limit, the efficiency of the T_1_ → S_1_ rev-ISC starts to decrease fast, and the TADF intensity goes down. At the same time, phosphorescence can appear as a visible contribution to the PL competing with TADF. The PL lifetime in this regime still increases upon cooling.

(iii) At the threshold temperature *T*_thr_, rev-ISC and TADF are quenched completely, and below this temperature the phosphorescence remains the only radiative decay mechanism. At *T*_thr_, the luminescence lifetime becomes equal to the intrinsic phosphorescence lifetime *τ*_Ph_ and is not changing with further cooling.

**Fig. 3:**
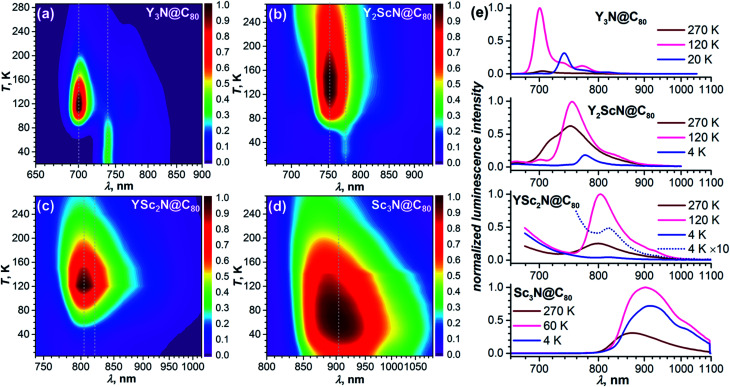
Temperature dependence of the luminescence spectra of (a) Y_3_N@C_80_, (b) Y_2_ScN@C_80_, (c) YSc_2_N@C_80_, and (d) Sc_3_N@C_80_ measured in polystyrene films between room temperature and 4 K; dotted lines mark the position of the peaks assigned to fluorescence and phosphorescence, the intensity scale is normalized separately for each compound. (e) Luminescence spectra for all four compounds at three selected temperatures (270 K; the temperature at which PL has the highest intensity; the lowest temperature studied).

**Fig. 4:**
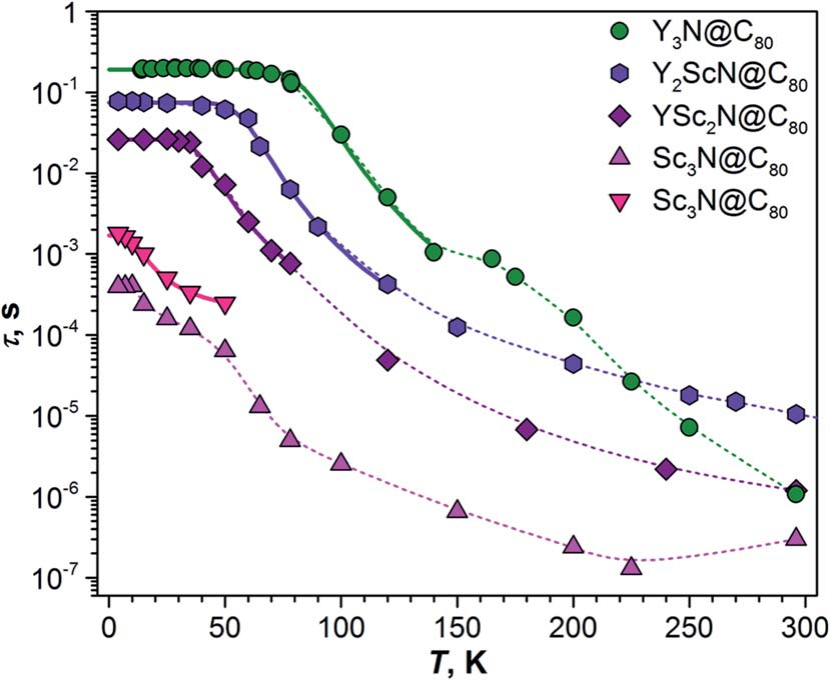
Luminescence lifetimes of Y_*x*_Sc_3−*x*_N@C_80_ compounds measured in polystyrene film at different temperatures. For Sc_3_N@C_80_, two sets of lifetimes are determined below 60 K. Thin dashed lines are shown to guide the eye, thick solid lines are fits of the low-temperature part based on eqn (1).

As TADF is characterized by strong changes of emission intensity and PL lifetime with temperature, we performed variable-temperature (VT) PL studies of Y_*x*_Sc_3−*x*_N@C_80_ compounds. The VT-PL spectra measured during the heating of the samples from helium to room temperatures are presented in Fig. 3a–d in the form of 2D contour plots with colour-coded normalized intensity. Spectra at three selected temperatures (270 K, the temperature of the highest luminescence intensity, and the lowest-temperature used in the studies) are plotted in Fig. 3e. Luminescence lifetimes measured at different temperatures are listed in Tables S1–S4† and plotted in Fig. 4.

The VT-PL studies of Y_3_N@C_80_ reported by us earlier revealed three regimes outlined above.^51^ When cooling from room temperature down to 120 K, the PL peak of Y_3_N@C_80_ at 705 nm enhances its intensity approximately 20-fold, which is accompanied by a gradual shift to 701 nm and narrowing of the emission peaks. In this temperature range, TADF is the only emissive pathway. As temperature decreases below 120 K, the intensity of the PL peak at 701 nm starts to decrease. At the same time, a new feature appears at 739 nm. This behaviour is caused by a decrease of the rev-ISC efficiency, leading to a decrease of the delayed fluorescence yield and appearance of the phosphorescence (the new peak at 739 nm). At 60 K, the peak at 701 nm disappears completely, whereas the peak at 739 nm is not changing with further cooling. Note that the prompt fluorescence usually does not show a strong temperature dependence, and the complete disappearance of the Y_3_N@C_80_ fluorescence below 60 K indicates that the compound does not show prompt fluorescence.

The VT-PL evolution for Y_2_ScN@C_80_ and YSc_2_N@C_80_ is qualitatively similar to that of Y_3_N@C_80_. The shoulder at 716 nm in the PL spectrum of Y_2_ScN@C_80_ decreases with cooling and essentially disappears by 120 K, whereas the intensity of the peak at 750 nm increases and reaches the maximum at 120–150 K. The increase of the luminescence intensity from RT to 120 K of *ca.* 80% is not as dramatic as for Y_3_N@C_80_. At further cooling, the peak at 750 nm decreases and disappears completely at 50 K, whereas a new feature at 775 nm becomes visible at 78 K first as a shoulder, and then as the only remaining PL peak below 50 K. The intensity of this peak remains almost constant between 50 and 4 K. In analogy to Y_3_N@C_80_ and based on the PL lifetimes (see below), we assign the peak at 775 nm to the phosphorescence and the peak at 750 nm to the delayed fluorescence of Y_2_ScN@C_80_. PL intensity of YSc_2_N@C_80_ near 800 nm also increases with cooling and reaches the maximum around 120 K, when its intensity becomes about 4 times higher than at room temperature. The band position is shifting gradually from 799 nm at RT to 804 nm at 120 K. Below 120 K, the PL intensity decreases, but the peak at 804 nm remains detectable down to 30–20 K. Only at the lowest temperatures, a new weak emission feature at 821 nm assigned to the phosphorescence of YSc_2_N@C_80_ can be distinguished. Its intensity is ≈100 times lower than that of the 804 nm peak at 120 K.

Sc_3_N@C_80_ shows a different thermal evolution of the PL spectra. Upon cooling, we observed an increase in the intensity and gradual shift of the peak position from 869 nm at RT to 904 nm at 60 K. The peak has an asymmetrical shape indicating either a complicated vibronic structure or coexistence of several emitting states with similar energy. The PL intensity increases threefold from RT to 60 K and then decreases to lower temperatures. Below 60 K, the broad PL peak changes its shape with the low-energy shoulder at 915 nm gaining in intensity, but the spectrum remains broad and unresolved even at the lowest temperature.

### Luminescence lifetimes and S_1_–T_1_ gap

The temperature dependence of luminescence lifetimes shown in Fig. 4 has a similar pattern for three Y_*x*_Sc_3−*x*_N@C_80_ molecules (*x* = 1–3) and confirms the TADF mechanism. The lifetime increases from 1–10 μs at RT to tens of ms below 100 K (Fig. S1 and Tables S1–S4†). At the threshold temperature *T*_thr_, the lifetime is levelled off and no further changes were observed at lower temperatures. The threshold temperature decreases with the increase of the Sc content in the endohedral cluster from 60 K for Y_3_N@C_80_, to 40 K for Y_2_ScN@C_80_, and 30 K for YSc_2_N@C_80_. Likewise, the luminescence lifetime at the plateau also decreases from 192 ms in Y_3_N@C_80_, 78 ms in Y_2_ScN@C_80_, to 26 ms in YSc_2_N@C_80_. These lifetimes can be interpreted as intrinsic phosphorescence lifetimes, *τ*_Ph_.

The key TADF parameter, the S_1_–T_1_ energy gap (*Δ*_ST_), can be determined as the energy difference of the fluorescence and phosphoresce peak maxima (marked with white dashed lines in Fig. 3a–c), giving 0.09 eV for Y_3_N@C_80_, 0.05 eV for Y_2_ScN@C_80_, and 0.03 eV for YSc_2_N@C_80_. Independent estimation of *Δ*_ST_ is possible from the temperature dependence of luminescence lifetimes using eqn (1):^65^1
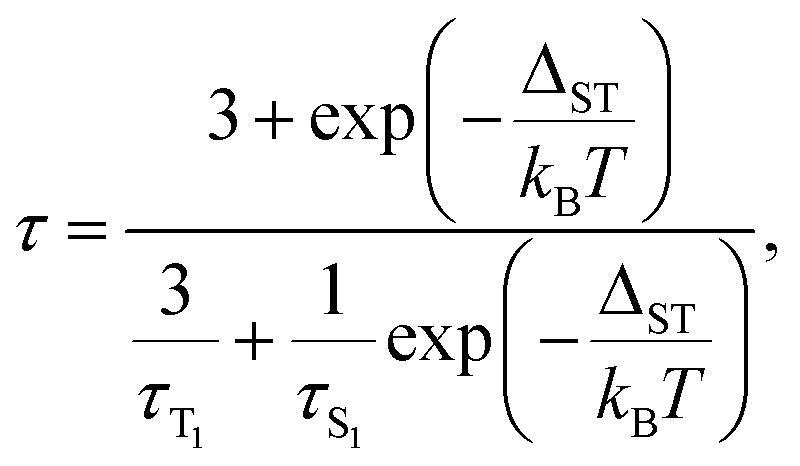
where *τ*_T_1__ and *τ*_S_1__ are intrinsic phosphorescence and prompt fluorescence lifetimes, respectively. Eqn (1) does not describe the higher-temperature part well, presumably because of additional processes in the polystyrene matrix and complicated dynamics of the endohedral units. But near *T*_thr_, the temperature dependence of the PL lifetimes is closely reproduced by eqn (1) and can be used to determine *Δ*_ST_. The *Δ*_ST_ values obtained by fitting experimental data with eqn (1) (Fig. 4) are close to those estimated from the peak positions (Table 1) confirming the reliability of the determined S_1_–T_1_ energy gaps.

The temperature dependence of the Sc_3_N@C_80_ luminescence lifetimes is more complex. Upon cooling from room temperature, the lifetime first decreases from 0.3 μs at 296 K to 0.13 μs at 225 K, but then shows a gradual increase to 13 μs at 65 K. Below this temperature, the decay of the PL intensity after the laser pulse has a double-exponential shape giving two sets of lifetimes (Fig. 4). In both sets the values continue to increase at cooling, giving lifetimes of 0.4 and 1.8 ms near 4 K. Levelling off at the plateau similar to other Y_*x*_Sc_3−*x*_N@C_80_ molecules is not observed. Considering that the lifetimes, *Δ*_ST_ gaps, and the threshold temperature *T*_thr_ decrease systematically with the increasing number of Sc atoms in the cluster, we suggest that the *Δ*_ST_ gap in Sc_3_N@C_80_ is very small, and TADF remains efficient even at 4 K. Decrease of the PL intensity below 60 K indicates that the efficiency of the T_1_ → S_1_ rev-ISC decreases here as well, but cooling below the lowest temperature accessible with our setup would be needed to freeze this process completely. In the absence of a clear transition between TADF and phosphorescence in the available temperature range, the *Δ*_ST_ gap of Sc_3_N@C_80_ cannot be determined experimentally. However, we can speculate that it is smaller than 0.03 eV obtained for YSc_2_N@C_80_.

Another feature of Sc_3_N@C_80_ not found in other Y_*x*_Sc_3−*x*_N@C_80_ molecules is the appearance of the fast radiative decay channel below 200 K with the lifetime of 8.8 ns, which decreases to 1.4 ns at 4 K. The assignment of this low-yield process remains open. A nanosecond timescale is typical for a prompt fluorescence. However, a transient absorption study of Sc_3_N@C_80_ showed that the S_1_ → T_1_ intersystem crossing is very fast and occurs within only 48 ps,^34,37^ which leaves no possibility for a nanosecond-long prompt fluorescence.

### Computational studies

The photophysical study of Y_*x*_Sc_3−*x*_N@C_80_ molecules revealed several systematic trends in the optical properties associated with the cluster composition. An increase of the number of Sc atoms leads to a modest red shift of the absorption and a much more pronounced red shift of the luminescence, a strong increase of the Stokes shift, a decrease of the S_1_–T_1_ gap, and a systematic decrease of luminescence lifetimes. To find the connection between these phenomena and the molecular and electronic structure of the metallofullerenes, detailed DFT modelling was performed.

**Fig. 5:**
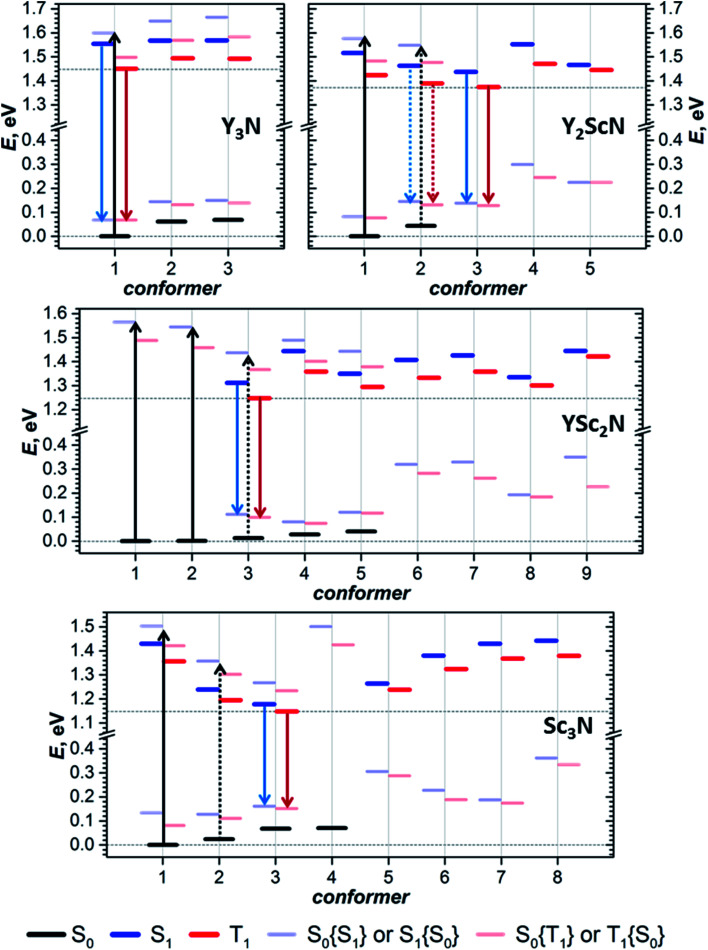
(TD)-DFT-computed relative energies of Y_*x*_Sc_3−*x*_N@C_80_ conformers in S_0_, S_1_, and T_1_ states. Thick black, blue, and red lines denote the energies of S_0_, S_1_, and T_1_ states, respectively, in their optimized geometries; thin pale blue lines are the energies of S_0_ states in optimized geometry of S_1_ state (S_0_{S_1_}) and S_1_ state in the geometry of S_0_ state (S_1_{S_0_}); thin pale red lines are S_0_{T_1_} or T_1_{S_0_} energies. Black arrows mark vertical excitations from the conformers with the lowest-energy S_0_ states, which should have the strongest contribution to the light absorption. Blue and red arrows denote transitions from the lowest-energy S_1_ and T_1_ states, corresponding to fluorescence and phosphorescence, respectively.

Preferable orientations of the M_3_N cluster inside the fullerene cage, which we will refer to as conformers, can vary substantially with the electronic state of the molecule. For instance, the lowest energy conformer of the Sc_3_N@C_80_ anion is different from that of the neutral molecule.^16,66^ To find the most preferable conformers, we used Fibonacci sampling^67^ to generate a set of 120 starting geometries with different cluster orientations for each Y_*x*_Sc_3−*x*_N@C_80_ molecule. All these structures were then optimized in the S_0_ and T_1_ electronic states at the PBE level using the Priroda code.^68,69^ Unique T_1_ conformers were then re-optimized in the S_1_ state using the time-dependent (TD)-DFT approach. In the course of TD-DFT optimization, the structures of S_1_ conformers remained very similar to corresponding T_1_ structures, and we will not distinguish them in the following discussion. Single point energy calculations at the PBE/def2-TZVPP level with ZORA scalar-relativistic corrections were then performed for all unique conformers in S_0_, S_1_, and T_1_ states using the ORCA suite.^70–76^ To treat S_1_ and T_1_ states at the same theoretical level, their energies were computed with TD-DFT. For estimation of vertical transition energies, the energies of S_1_ and T_1_ states in S_0_ geometry and the energy of the S_0_ state in S_1_ and T_1_ geometries were also calculated. Computed energies are summarized in Fig. 5 and listed in Table S5.† The conformers are numbered first in ascending order according to their relative energies in the S_0_ state and are labelled in the further discussion as **conf N**. In particular, **conf 1** is always the lowest energy conformer for the ground electronic state. If the vibrational structure of the electronic transition is not resolved, the point of the highest intensity in the broad band corresponds to the vertical transition. For absorption, it will be denoted as S_0_ → S_1_{S_0_}, which means that the S_0_ and S_1_ energies are computed in the optimized geometry of the S_0_ state. Likewise, vertical transitions corresponding to fluorescence are denoted as S_1_ → S_0_{S_1_}, which means that the energies of S_1_ and S_0_ states are computed in the optimized S_1_ geometry. In the following discussion, the theoretical Stokes shift, *Δ*_Stokes_, is calculated as *E*(S_0_ → S_1_{S_0_}) − *E*(S_1_ → S_0_{S_1_}) for the lowest energy S_0_ and S_1_ conformers, whereas the adiabatic S_1_–T_1_ energy gap, *Δ*_ST_, is the energy difference of S_1_ and T_1_ states in their optimized geometries.

For Y_3_N@C_80_, computations revealed three conformers for the ground state and analogous conformers for excited states. **Conf 1** with *C*_3_ symmetry (Fig. 2) has the lowest energy in both S_0_ and S_1_/T_1_ states, which means that Y_3_N@C_80_ retains its structure upon photoexcitation. Relative energies of **conf 2** and **conf 3** in the ground state are 0.062 and 0.069 eV, and these conformers may also contribute to the absorption spectra at room temperature. Adiabatic S_1_ ↔ S_0_ excitation is predicted at 1.554 eV, whereas vertical transitions S_0_ → S_1_{S_0_} and S_1_ → S_0_{S_1_} are predicted at 1.599 and 1.487 eV, respectively, giving *Δ*_Stokes_ of 0.112 eV. The computed *Δ*_ST_ gap is 0.104 eV, very close to the experimental estimation near 0.09–0.10 eV (Table 1).

For Y_2_ScN@C_80_, we found two S_0_ conformers with the energy difference of 0.044 eV and their counterparts for S_1_/T_1_ states, but also three other S_1_/T_1_ conformers without analogous structures in the ground state. **Conf 1** resembles the *C*_3_-symmetric structure of Y_3_N@C_80_ with one Y replaced by Sc. Adiabatic and vertical S_0_ → S_1_ excitations for **conf 1** at 1.516 and 1.576 eV, respectively, are very close to the Y_3_N@C_80_ values in line with the similarity of the absorption spectra (Fig. 2). **Conf 3** has the lowest energy in the S_1_/T_1_ excited states (1.438 eV for S_1_), and its vertical S_1_ → S_0_{S_1_} transition has an energy of 1.300 eV. Thus, computed *Δ*_Stokes_ is 0.276 eV, and its considerable increase in comparison to Y_3_N@C_80_ is caused by switching from **conf 1** in the ground state to **conf 3** in the excited state. The *Δ*_ST_ gap in **conf 3** is 0.063 eV, again in good agreement with the experimental value of ≈0.05 eV. Note that **conf 2** of Y_2_ScN@C_80_ is only slightly higher in energy than **conf 3** in the excited state and hence may also contribute to the luminescence. But its transition energy and *Δ*_ST_ gap are very similar to the values of **conf 3**.

YSc_2_N@C_80_ has the most diverse manifold of conformers in the whole Y_*x*_Sc_3−*x*_N@C_80_ series. We found 5 ground state conformers and 7 excited state conformers, of which only 3 have corresponding S_0_ structures. **Conf 1** and **conf 2** are equally stable and have similar S_0_ → S_1_{S_0_} energies of 1.564 and 1.544 eV, respectively. At the same time, three other S_0_ conformers also have close relative energies within 0.041 eV, but smaller excitation energies (down to 1.402 eV for **conf 5**). All five S_0_ conformers may contribute to the absorption spectrum at room temperature, causing broadening and red shift when compared to the spectra of Y_3_N@C_80_ and Y_2_ScN@C_80_. The lowest energy S_1_/T_1_ conformer of YSc_2_N@C_80_ is **conf 3** with an S_1_ → S_0_{S_1_} transition at 1.201 eV and *Δ*_ST_ gap of 0.062 eV. Rearrangement from **conf 1** in S_0_ into **conf 3** in the S_1_ state leads to a rather large value of the computed *Δ*_Stokes_, 0.363 eV.

Sc_3_N@C_80_ also shows a rich structural variability with 4 S_0_ and 7 S_1_/T_1_ conformers. S_0_ conformers coincide with those found in our earlier computational study,^16^ which used a less extended set of starting configurations. **Conf 1** and **conf 2** have close relative energies in the ground state, but considerably different energies of S_0_ → S_1_{S_0_} transitions at 1.503 and 1.333 eV, respectively. Thus, calculations predict a further red shift of absorptions in comparison to other Y_*x*_Sc_3−*x*_N@C_80_ molecules and broadening of the spectral profile, in good agreement with experimental observations. **Conf 3** is the lowest energy conformer in the S_1_ excited states, followed by **conf 2** at 0.025 eV higher. The S_1_ → S_0_{S_1_} transitions of **conf 3** and **conf 2** occur at 1.017 and 1.123 eV, respectively, giving further red shift of the emission and the *Δ*_Stokes_ value of 0.486 eV. The calculated *Δ*_ST_ gap in **conf 3** is only 0.030 eV.

The structural flexibility of M_3_N@C_80_ molecules associated with the motion of the endohedral cluster^16,66^ leads to a possibility of switching between conformers when the thermal motion of the cluster is frozen at low temperatures.^17–20^ Our work proves that this conformational flexibility indeed appears in the excited states. Each of the Y_*x*_Sc_3−*x*_N@C_80_ molecules we studied has several conformers with close energies in both S_0_ and T_1_/S_1_ states. Since conformers have significantly different spectroscopic properties, as can be concluded from the different S_0_ ↔ S_1_ transition energies discussed above, this conformational variability should have a profound influence on the optical spectra. The changes in the spectral shape and shift of the peak positions at lower temperatures can be rooted in the changes in the conformer composition. For example, the shoulder at higher energy in the emission spectrum of Y_2_ScN@C_80_ and its disappearance at a lower temperature may be caused by a higher-energy conformer, whose thermal population decreases during cooling. The gradual shift of the Sc_3_N@C_80_ emission to longer wavelengths upon cooling may be also rooted in a similar variation of conformational composition. A more definitive answer is hardly possible at this moment because the spectra have unresolved vibronic structure, which can also change with temperature (for instance, by freezing out hot transitions).

Switching between conformers driven by their thermodynamic stability requires equilibration of the system. Since emission takes place on a relatively long timescale of μs and ms, and since barriers for reorganization between conformers are expected to be rather low,^9,16,17^ we believe that thermal equilibrium between conformers in the T_1_ state is established between excitation and emission and that the emission proceeds with the lowest energy conformers of the triplet state. Excess energy which may be needed to overcome the barriers between conformers is granted by the internal conversion, which follows after the molecule is excited by a photon to some higher energy electronic state (Fig. 1). Equilibration in the ground state is more questionable, and the system can stay in the non-equilibrium conformer distribution after emission. Morton *et al.* reported that ErSc_2_N@C_80_ changes conformer composition when irradiated by a 532 nm laser at 5 K, and that annealing at 30 K in the dark restores the original composition.^19^ Magnetic studies of HoLu_2_N@C_80_ and TbSc_2_N@C_80_ also showed that rotation of the endohedral cluster is frozen near 50 K.^18^ Thus, likely, the conformer distribution of Y_*x*_Sc_3−*x*_N@C_80_ molecules in the ground electronic state is also not completely restored in our experiments below 30 K. For instance, the population of **conf 3** can be enhanced in the S_0_ state of YSc_2_N@C_80_ and Sc_3_N@C_80_ after continuous irradiation. But our photoluminescence studies cannot follow the ground state conformer composition, whereas results for the excited states will not be affected by this effect.

**Fig. 6:**
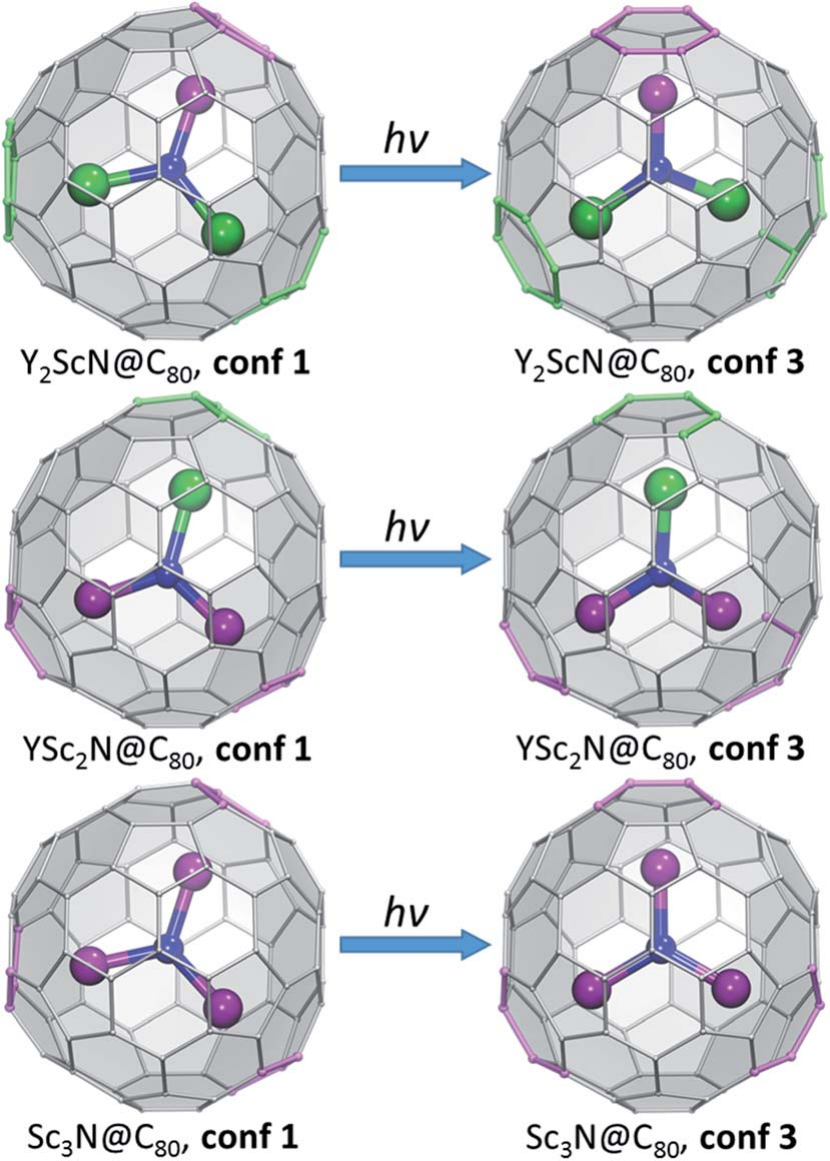
Switching between the lowest-energy conformers of S_0_ (**conf 1**) and S_1_/T_1_ (**conf 3**) electronic states in Y_2_ScN@C_80_, YSc_2_N@C_80_, and Sc_3_N@C_80_. Carbon atoms with Y–C distance less than 2.6 Å and Sc–C distance less than 2.5 Å are highlighted in green and magenta, respectively. The fullerene cage is shown in the same orientation in all figures. **Conf 1** has *C*_3_ symmetry in Sc_3_N@C_80_ and Y_3_N@C_80_ (not shown here, see Fig. 2), and a similar cluster orientation is also realized in YSc_2_N@C_80_ and Y_2_ScN@C_80_. In **conf 3** of Sc_3_N@C_80_, the cluster is rotated around the *C*_3_-axis to adopt *C*_3v_ symmetry. For YSc_2_N and Y_2_ScN, the analogous cluster position inside the cage is not stable because of the larger cluster size.

To summarize, our computations show that only in Y_3_N@C_80_ the molecular structure is not changing in the excited state. The lowest energy conformers of Y_2_ScN@C_80_, YSc_2_N@C_80_, and Sc_3_N@C_80_ molecules are different in the ground (S_0_) and excited (S_1_/T_1_) electronic states, which results in the photoswitching. The metal cluster in these molecules rotates upon photoexcitation to adopt a lower energy configuration (Fig. 6). The S_0_ → S_1_{S_0_} excitation energy of the most stable ground state conformer (**conf 1**) is weakly affected by the cluster composition in the whole Y_*x*_Sc_3−*x*_N@C_80_ series, decreasing by 0.098 eV from Y_3_N@C_80_ to Sc_3_N@C_80_. On the other hand, the S_1_ → S_0_{S_1_} transition energy for the most stable exited-state conformer decreases in the same row by as much as 0.470 eV. Altogether, this results in a considerable increase in the difference between absorption and emission energies for Y_*x*_Sc_3−*x*_N@C_80_ molecules with the decrease of *x*. These computational findings are in perfect agreement with the spectroscopic observations (Fig. 2 and 3) and confirm the switching of the endohedral cluster position in the excited state.

**Fig. 7:**
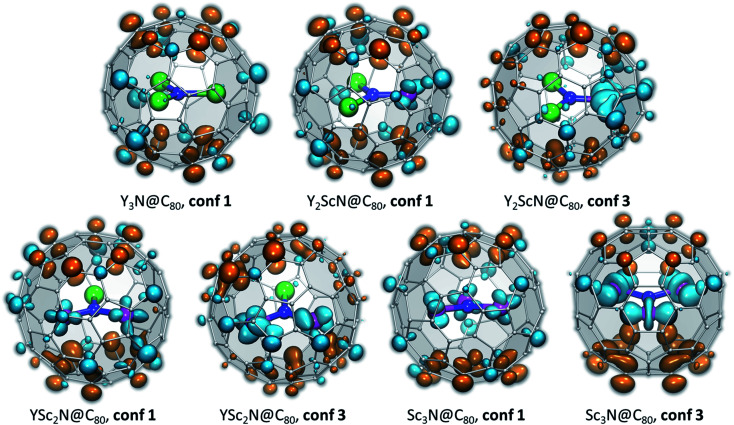
Difference electron charge density Δ*ρ*_el_(S_0_ → S_1_) = *ρ*_el_(S_1_) − *ρ*_el_(S_0_) computed with TD-DFT for the lowest-energy S_0_ and S_1_/T_1_ conformers of Y_*x*_Sc_3−*x*_N@C_80_. Blue lobes denote areas where the electron density is accumulated in the excited state, dark orange lobes mark areas where the electron density is depleted upon excitation. If the excited state is described as a formation of the electron–hole pair (exciton), blue lobes in Δ*ρ*_el_(S_0_ → S_1_) isosurfaces illustrate the spatial localization of the electron, and orange lobes describe (de)localization of the hole. The isosurfaces are visualized with VMD.^77^

To understand what may be the reason behind the change of the endohedral cluster position in the excited state, we analyzed frontier molecular orbitals, spin densities in the triplet state (*ρ*_spin_(T_1_)), and the difference electron charge density (*ρ*_el_) for the S_0_ → S_1_ excitation (Δ*ρ*_el_(S_0_ → S_1_) = *ρ*_el_(S_1_) − *ρ*_el_(S_0_)). The difference density gives the most informative description of the changes in the electronic structure upon excitation, and Δ*ρ*_el_(S_0_ → S_1_) isosurfaces for the key conformers of Y_*x*_Sc_3−*x*_N@C_80_ molecules are plotted in Fig. 7, whereas frontier MOs and spin densities are shown in ESI (Fig. S2 and S3).† In Δ*ρ*_el_(S_0_ → S_1_) plots, blue lobes denote areas where the electron charge density is accumulated in the excited state, whereas dark orange lobes mark areas where the electron charge density is depleted. Since S_0_ → S_1_ excitations in the studied molecules have mainly HOMO → LUMO character, Δ*ρ*_el_(S_0_ → S_1_) is approximately equal to the difference of the orbital densities of the LUMO and HOMO, and hence the blue and orange lobes in Fig. 7 reflect the spatial localization of the LUMO and HOMO, respectively. On the other hand, *ρ*_spin_(T_1_) has a similar shape to Δ*ρ*_el_(S_0_ → S_1_), but has the same sign almost everywhere and therefore is less informative. In the discussion below, we will refer to the belt connecting metal-coordinated fragments of the fullerene cage as the equator, whereas the parts of the fullerene above and below the plane of the M_3_N cluster as poles.

In the M_3_N@C_80_ molecules with M = Y and other heavy metals (but not Sc), the HOMO and LUMO are localized on the poles and equator, respectively. Therefore, photoexcitation results in the electron transfer from the poles to the equator, as can be well seen in the Δ*ρ*_el_(S_0_ → S_1_) isosurface plot for Y_3_N@C_80_, **conf 1** (Fig. 7). Metal contributions to the difference density are very small and hardly seen. The situation changes when a Sc atom replaces Y. The HOMO of Y_2_ScN@C_80_ (**conf 1**) is still localized on the poles and the LUMO on the equator, but the latter also has a noticeable Sc contribution, which results in a partial localization of Δ*ρ*_el_(S_0_ → S_1_) on Sc. The same pattern is observed in Δ*ρ*_el_(S_0_ → S_1_) isosurfaces of YSc_2_N@C_80_ and Sc_3_N@C_80_, which show that each Sc atom has a contribution in the LUMO (hence blue lobes on Sc atoms in Fig. 7), but has a little effect on the HOMO. Another immediate conclusion from the analysis of Δ*ρ*_el_(S_0_ → S_1_) isosurfaces in Fig. 7 is that in all Y_*x*_Sc_3−*x*_N@C_80_ molecules (*x* = 0–2), localization of the excess charge density on Sc atoms in the lowest-energy T_1_ conformer (**conf 3**) is substantially larger than in the ground state conformers (**conf 1**).

Thus, Δ*ρ*_el_(S_0_ → S_1_) isosurfaces show that in Sc-containing M_3_N@C_80_ molecules the first excitation has a considerable charge-transfer character. In analogy to ligand-to-metal charge transfer (LMCT) in the photophysics of coordination compounds, this kind of excitation can be called “fullerene-to-metal charge transfer” (FMCT). The FMCT character of the photoexcitation increases incrementally with the number of Sc atoms in Y_*x*_Sc_3−*x*_N@C_80_ molecules and is further enhanced in those conformers of Y_*x*_Sc_3−*x*_N@C_80_, which are stabilized in the excited state. The maximization of the excess charge density on Sc atoms appears to be a driving force for the cluster rearrangement in the excited state.

The spatial separation of the HOMO and LUMO over the fullerene cage leads to the relatively small *Δ*_ST_ gap in Y_3_N@C_80_ and enables efficient TADF.^51^ Enhancement of the spatial separation of the frontier orbitals due to the adherence of the LUMO to Sc atoms and the increase of the FMCT character of the first excitation across the Y_*x*_Sc_3−*x*_N@C_80_ series is the reason for an even further decrease of the *Δ*_ST_ gap in the Y_*x*_Sc_3−*x*_N@C_80_ molecules as *x* decreases from 3 to 0. And since the FMCT character is enhanced in excited-state conformers such as **conf 3** (Fig. 7), these conformers also feature the smallest *Δ*_ST_ gaps (Fig. S4†). To summarize, preferential localization of the excess electron charge and spin density in the excited state on Sc atoms and further enhancement of this effect in the conformers stabilized in the excited states leads to a decrease of the *Δ*_ST_ gaps for these structures, which agrees perfectly with our spectroscopic results (Table 1). In Sc_3_N@C_80_ this effect is so strong that the *Δ*_ST_ gap is below the limit which can be determined in our experiments.

### Photoelectron spectroscopy (PES) of the Sc_3_N@C_80_^−^ anion

The lowest-energy T_1_-conformer of Sc_3_N@C_80_ from this work, **conf 3**, has the same *C*_3v_-symmetric arrangement of the Sc_3_N cluster inside fullerene as was earlier found in the lowest energy conformer of the Sc_3_N@C_80_^−^ anion.^16,66^ Further comparison shows that there is indeed a close parallelism between optimal conformers for the neutral fullerene in the T_1_ excited state and the monoanion (see Table S6† for comparison of conformer energies). Similar to the triplet state of Sc_3_N@C_80_, the Sc_3_N@C_80_^−^ monoanion also has a much higher spin density on the cluster in **conf 3** (*C*_3v_ conformer) than in **conf 1** (*C*_3_ configuration). Thus, the structure with the largest spin population on the endohedral cluster has the lowest energy both in the anionic state and the triplet excited state, which leads to a similar structural rearrangement upon negative charging or photoexcitation of a Sc_3_N@C_80_ molecule.

**Fig. 8:**
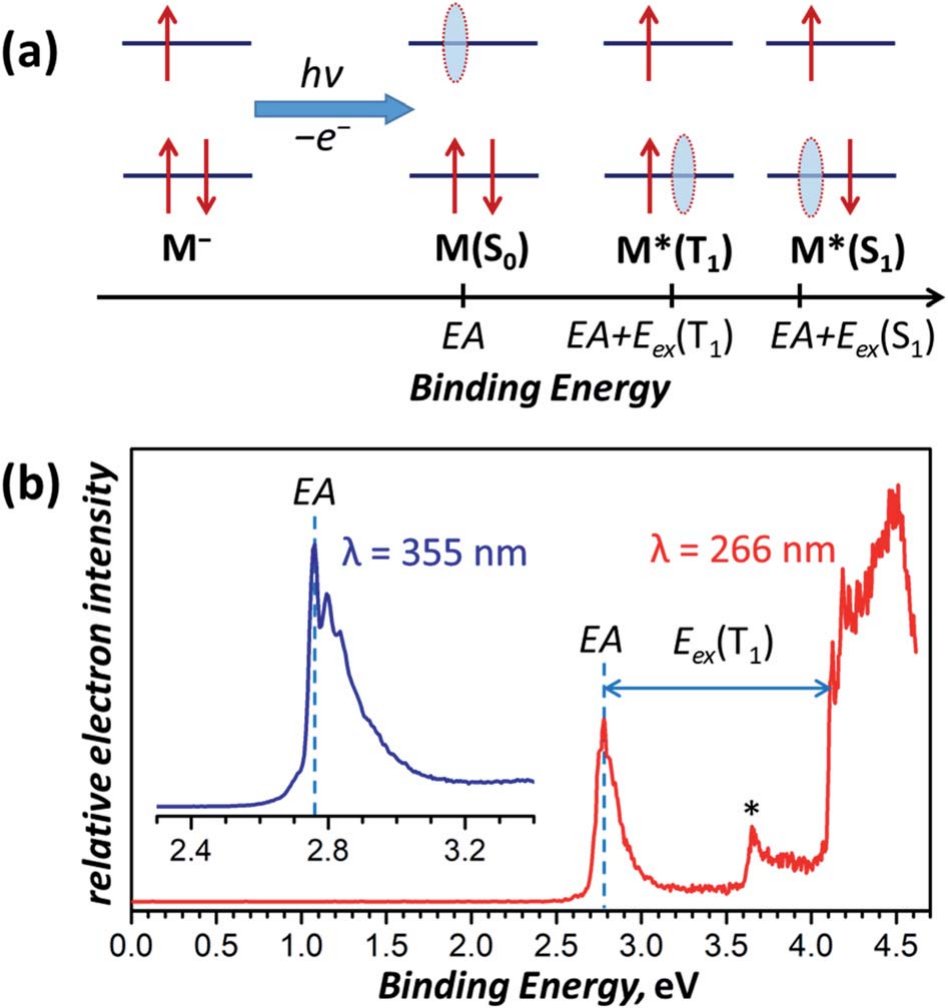
(a) Schematic description of the electron photoemission process with an anion **M−** resulting in a neutral molecule **M** in different electronic states (S_0_, T_1_, S_1_, *etc.*) and correspondence of the binding energies with the molecular electronic properties, such as the electron affinity (EA) and excitation energies S_0_ → T_1_ and S_0_ → S_1_ denoted as *E*_ex_(T_1_) and *E*_ex_(S_1_), respectively; red arrows denote electrons, whereas light blue ovals show “holes” left behind the photo-emitted electrons. (b) Photoelectron spectrum of Sc_3_N@C_80_^−^ measured at 12 K with laser excitations at 355 nm (3.49 eV, inset) and 266 nm (4.66 eV). The asterisk denotes an auto-detachment peak similar to that observed in PES spectra of C_60_^−^.^88,89^

We can establish a further connection between anion and triplet state geometries of Sc_3_N@C_80_ by a low-temperature photoelectron spectroscopic (PES) study^78^ of the Sc_3_N@C_80_^−^ anion. In the PES experiment, laser photoionization of the anion **M−** cooled to helium temperature results in the ejection of an electron with a certain binding energy. Fig. 8a summarizes the photoelectron ejection mechanisms possible in the low energy range. Electron ejection from the SOMO of the anion leaves the molecule **M** in the ground electronic state, and the binding energy for this process is equal to the electron affinity (EA) of the molecule. However, an electron can be also ejected from the low-lying two-fold occupied orbital, which will give either the triplet or the singlet excited state of the neutral molecule, M*. The binding energy, in this case, corresponds to the sum of EA and electronic excitation energy *E*_ex_ of the neutral molecule.

The photoelectron spectrum of Sc_3_N@C_80_^−^ in Fig. 8b gives the EA of 2.76 ± 0.01 eV, which agrees well with an earlier estimation of EA = 2.81 ± 0.05 eV from the high-temperature ion-molecular equilibrium analyzed by Knudsen-cell mass spectrometry.^79^ To our knowledge, this is the first determination of the EMF electron affinity by high-resolution PES, although the method had been widely used for EA measurements of empty fullerenes and their derivatives.^80–86^ For comparison, the most accurate EA of C_60_ determined by a similar technique is 2.684 ± 0.001 eV.^87^ Since Sc_3_N@C_80_^−^ is cooled down to 12 K and equilibrated before the photoionization, it is reasonable to assume that the most intense feature in the PES spectrum corresponds to the Sc_3_N@C_80_^−^ → Sc_3_N@C_80_ process of the *C*_3v_ conformer (**conf 3**) in both anion and neutral states. Photoionization yielding lower-energy conformers of the neutral Sc_3_N@C_80_ should give somewhat smaller EA but is expected to have much lower intensity by the Franck–Condon principle and may be responsible for the tail of the main peak at 2.60–2.75 eV.

The PES peak at binding energy above 4 eV corresponds to the T_1_ excited state of Sc_3_N@C_80_. The large width and complex structure of the peak are likely to be caused by the distribution of conformers, vibronic transitions, and overlap with the S_1_ state, which should appear at essentially the same energies because of the small *Δ*_ST_ gap. The lowest energy peak likely to correspond to the **conf 3** in the T_1_ state, which yields 1.35 eV as the *E*_ex_(S_0_ → T_1_) transition energy of the **conf 3**. This value is very close to the emission energy of Sc_3_N@C_80_ determined in low-temperature PL studies (Table 1).

### Light-induced EPR spectroscopy

The fullerene-to-metal charge transfer in the S_1_/T_1_ excited states of Y_*x*_Sc_3−*x*_N@C_80_ molecules implies that the spin density in the T_1_ states has large Sc contribution, which should lead to strong hyperfine interactions. Spin density in the Sc_3_N@C_80_^−^ anion is mainly localized on the endohedral cluster resulting in a large ^45^Sc hyperfine coupling constant of 5.3–5.6 mT (≈150 MHz).^90–93^ If the analogy between triplet state and anion holds, enhanced hyperfine constants for Sc atoms are expected in the T_1_ state of Y_*x*_Sc_3−*x*_N@C_80_. To study the electronic structure and the dynamics of the triplet states we have employed light-induced pulsed W-band EPR spectroscopy (94 GHz/3.4 T). Experiments were performed in toluene-*d*_8_ solutions frozen at 20 K to ensure sufficiently long T_1_ lifetimes.

**Fig. 9:**
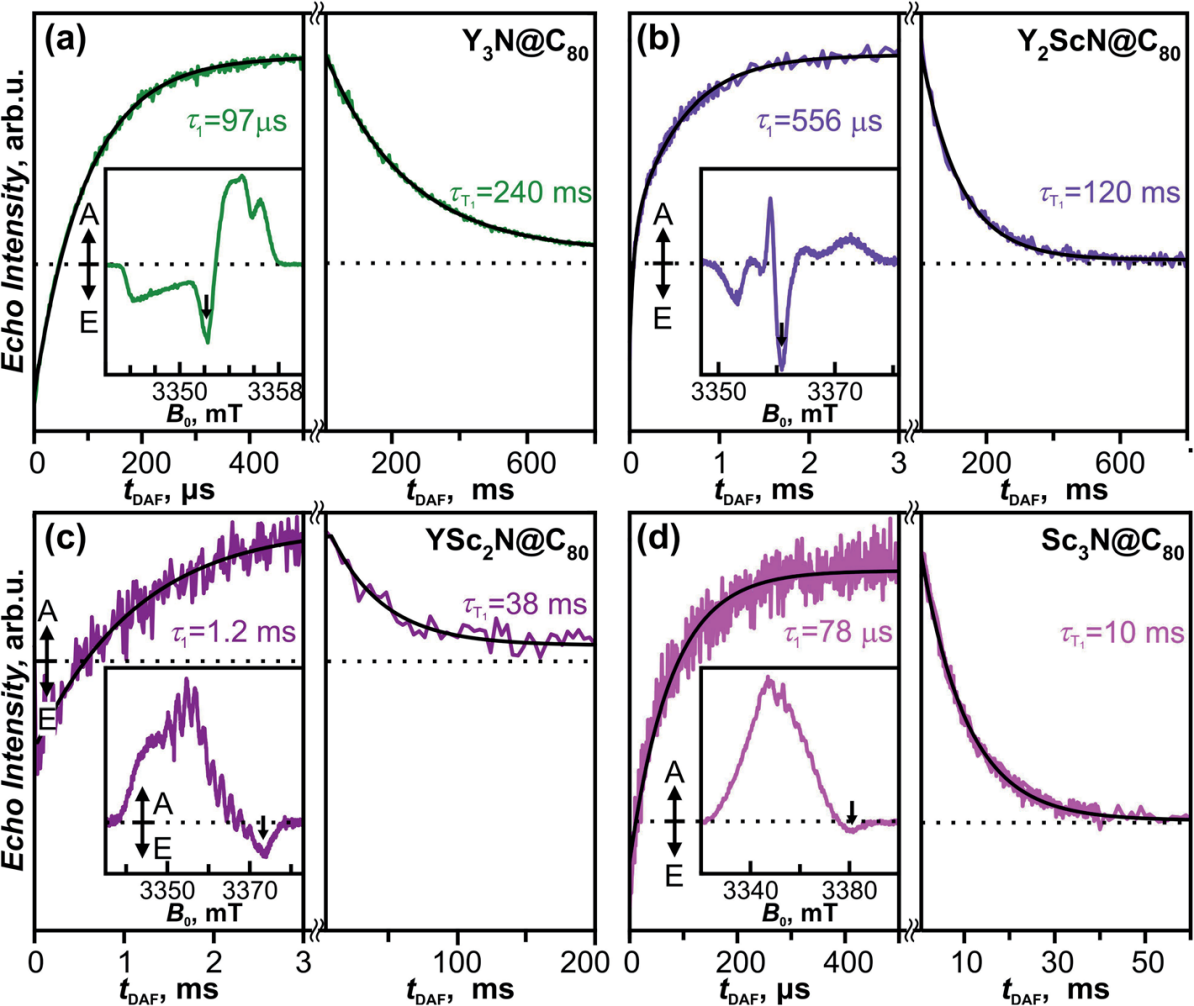
Insets: W-band time-resolved electron spin echo (ESE) detected EPR spectra of the (a) Y_3_N@C_80_, (b) Y_2_ScN@C_80_, (c) YSc_2_N@C_80_ and (d) Sc_3_N@C_80_ excited triplet states (*S* = 1) in a frozen toluene-*d*_8_ matrix at 20 K recorded at *t*_DAF_ = 500 ns after the laser flash at 488 nm. Main figure: ESE intensity of the photoexcited triplets as a function of *t*_DAF_ (arrows in the insets mark the field position of time trace recordings). Directly after the laser flash, the polarization of the EPR signal decays with the spin–lattice relaxation time *τ*_1_. This process is seen as the increase of the ESE intensity because the emissive signal transforms into absorptive as the Boltzmann population of T_1_ components is restored. On a longer timescale, the ESE signal decays according to the triplet lifetime *τ*_T_1__. Black lines represent the exponential fits of the corresponding time traces, giving the *τ*_1_ and *τ*_T_1__ parameters displayed in the figure.

The field-swept Electron Spin Echo (ESE) detected W-band EPR spectra of the Y_*x*_Sc_3−*x*_N@C_80_ series, recorded upon laser excitation in frozen toluene-*d*_8_ at 20 K, are shown in Fig. 9 (insets) and Fig. S5.† Reference spectra measured in the dark showed no EPR signal, and the spectra recorded upon irradiation are therefore assigned to the corresponding excited triplet states. Directly after the laser flash (delay after flash time, *t*_DAF_, of 500 ns), the EPR spectra show spin-polarized character, featuring both absorption (A) and emission (E) lines (Fig. 9 insets). The polarization pattern stems from the non-equilibrium population of the triplet energy levels built during the intersystem crossing from the excited singlet state. A detailed analysis of the polarized EPR patterns is non-trivial due to the complex cluster conformational dynamics discussed above and is thus out of the scope of this work. The ESE signals evolve through two distinct time regimes as illustrated for the emissive line intensities in Fig. 9. During the first few hundreds of μs (or in the low ms range) after the laser flash, the polarized pattern diminishes due to the spin–lattice relaxation characterized by the *τ*_1_ time. The purely absorptive EPR signal formed after this first period (Fig. S5†) then corresponds to the Boltzmann population of the triplet energy levels. Its exponential decay during the next tens to hundreds of ms corresponds to the relaxation of the T_1_ to the S_0_ ground state and is characterized by the triplet lifetime *τ*_T_1__.

The *τ*_1_ times obtained for the Y_*x*_Sc_3−*x*_N@C_80_ series span over an order of magnitude from 78 μs for the Sc_3_N@C_80_ to 1.2 ms for YSc_2_N@C_80_. The trend shows no apparent correlation with the cluster composition and the difference between the shortest and the longest *τ*_1_ is rather surprising. The position of the emission feature within the polarized spectra varies considerably among the fullerene series, being close to the center for Y_3_N@C_80_ and Y_2_ScN@C_80_ and near the high-field edge for YSc_2_N@C_80_ and Sc_3_N@C_80_ excited triplets. Given the powder-like character of the EPR spectra, generally, the microwave pulse excitation closer to the center covers more molecular orientations than at the edge, where single crystal-like orientation-selected data can be achieved. If a significant spin–lattice relaxation anisotropy occurs and depends on the molecular orientations, the different positions within the spectrum may result in incompatible data. Thus, a more detailed study with different field positions is needed to yield insight into the relationship between the cluster composition and/or motion and relaxation dynamics, which is beyond the scope of this work.

The trend of the triplet lifetimes *τ*_T_1__ is more straightforward. At 20 K, *τ*_T_1__ decreases with the increasing number of Sc atoms in the cluster from 240 ms for Y_3_N@C_80_ to 10 ms for Sc_3_N@C_80_. This trend agrees with the results from the phosphorescence measurements, and the *τ*_T_1__ and *τ*_Ph_ values show a reasonable quantitative match (Table 1), if we keep the different experimental conditions in mind, particularly the different solvent matrix (frozen toluene-*d*_8_ in EPR *vs.* polystyrene in luminescence experiments).

**Fig. 10:**
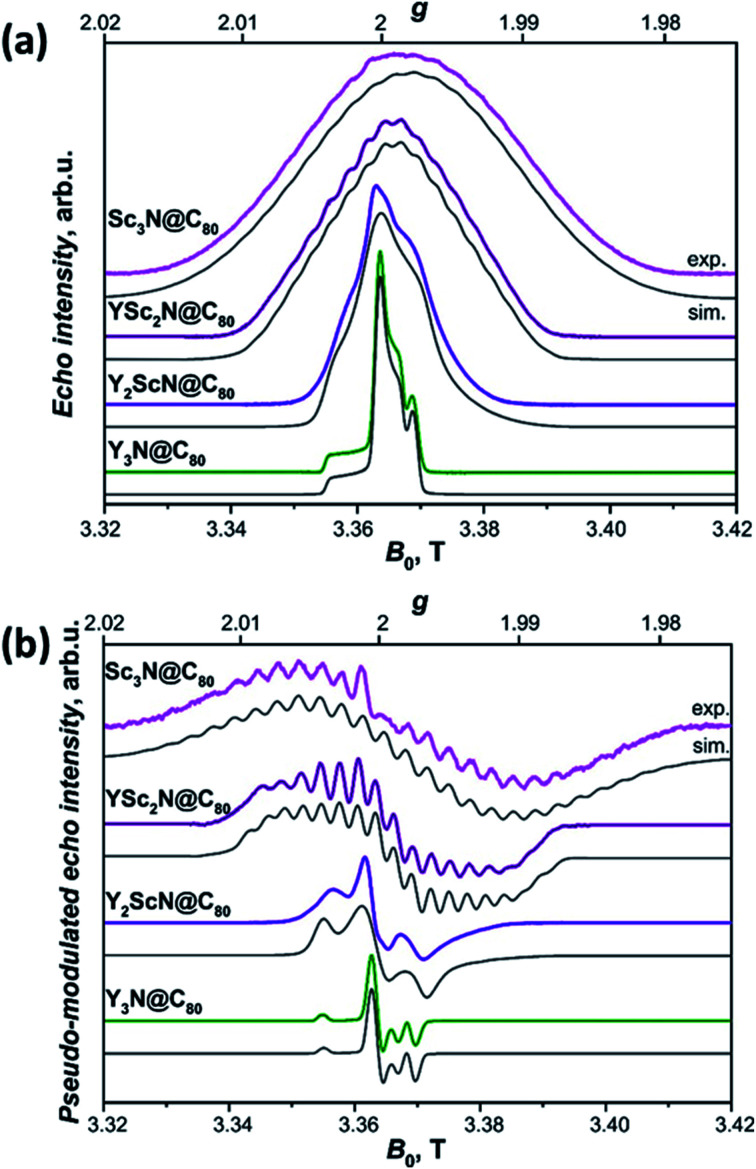
(a) W-band ESE-detected EPR spectra of the Y_3_N@C_80_ (green line), Y_2_ScN@C_80_ (violet line), YSc_2_N@C_80_ (purple line) and Sc_3_N@C_80_ (magenta line) excited triplet states recorded in frozen toluene-*d*_8_ solution at 20 K under continuous 510 nm laser illumination. Grey lines represent EPR spectra calculated using the spin Hamiltonian in eqn (2) and the magnetic parameters summarized in Table 2. (b) The same ESE EPR spectra processed by a field pseudo-modulation procedure (modulation amplitude of 1.5 mT) to resemble continuous-wave (CW) EPR spectra. The ^45^Sc hyperfine multiplets are clearly resolved in the YSc_2_N@C_80_ and Sc_3_N@C_80_ traces. Reduced spin density on Y atoms together with smaller nuclear *g*-factor (*g*_n_ = −0.27 for ^89^Y *versus g*_n_ = 1.36 for ^45^Sc) lead to much smaller ^89^Y hyperfine couplings, which cannot be resolved in the EPR spectra.

The long lifetimes of the Y_*x*_Sc_3−*x*_N@C_80_ triplets at 20 K provide an additional advantage for the light-induced EPR experiments. The spectra obtained under continuous irradiation (Fig. 10, compare with Fig. S5†) correspond to the equilibrium population of the triplet energy levels and are easier to analyze due to the absence of polarization effects. Besides, EPR signal acquisition is no longer limited by the laser pulse repetition rate, giving improved signal-to-noise ratio under continuous illumination. Already a brief inspection of the EPR spectra in Fig. 10 reveals several interesting trends. The increasing number of Sc atoms in the cluster leads to the broadening of the EPR spectra. Their central position moves towards lower *g* values and a regular signal splittings appear for YSc_2_N@C_80_ and Sc_3_N@C_80_. The splittings were already apparent in the polarized EPR spectra in Fig. 9 and S5.† They become best resolved when we process the absorption data from Fig. 10a using a pseudo-modulation procedure to resemble cw-like EPR spectra (Fig. 10b). The splittings indicate a significant hyperfine interaction between the triplet exciton (two unpaired electrons) and the ^45^Sc nuclear spin(s) (*I* = 7/2, natural abundance 100%). The broadening and the rather isotropic character of YSc_2_N@C_80_ and Sc_3_N@C_80_ EPR spectra most likely stems from a combined effect of the ^45^Sc hyperfine splitting and a larger zero-field splitting. Together with the shifts of the spectrum towards the *g* < 2 region these observations suggest a larger metal contribution to the spin-bearing orbitals as the cluster composition changes from Y_3_N to Sc_3_N.

Interpretation of the EPR spectra of triplets with strong hyperfine interactions and nuclear spin *I* > 1/2 requires a spin Hamiltonian (*Ĥ*_spin_), which includes electron Zeeman (*Ĥ*_EZ_), zero-field splitting (*Ĥ*_ZFS_), hyperfine (*Ĥ*_HFI_), nuclear Zeeman (*Ĥ*_NZI_), and nuclear quadrupole (*Ĥ*_NQI_) terms:2
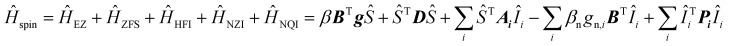
where the *i* sums run over all magnetically active nuclei, ***g*** and ***A***_***i***_ represent the *g*-tensor and hyperfine coupling tensors determined by the principal values in the corresponding principal axis systems, and *g*_n,*i*_ are nuclear *g*-factors.^94^ The zero-field splitting term in the principal frame transforms to:

3 *Ĥ*_ZFS_ = *Ŝ*^T^***D****Ŝ* = *D*[*Ŝ*_*z*_^2^ − *S*(*S* + 1)/3] + *E*(*Ŝ*_*x*_^2^ − *Ŝ*_*y*_^2^)

with *D* and *E* defined through the principal values of ***D*** as *D* = 3/2*D*_*z*_, and *E* = (*D*_*x*_ − *D*_*y*_)/2. The electric nuclear quadrupole term for each quadrupolar nucleus can be written as in eqn (4):^95^

4 *Ĥ*_NQI_ = *Î*^T^***P****Î* = *K*[3*Î*_*z*_^2^ − *I*(*I* + 1) + *η*(*Î*_*x*_^2^ − *Î*_*y*_^2^)]

with5
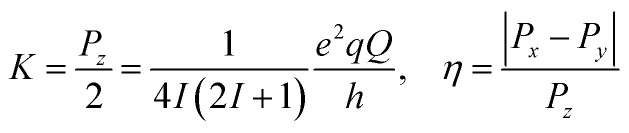
where *P*_*x*_, *P*_*y*_, and *P*_*z*_ are the diagonal elements of ***P***, *q* is the electric field gradient along the principal axis (*z*-axis) of the largest field gradient, *Q* is the nuclear quadrupole moment. The factor *e*^2^*qQ*/*h* is usually denoted as the quadrupolar coupling constant in MHz. The asymmetry parameter *η* is a measure of the electric field gradient asymmetry in the plane perpendicular to the *z*-axis and may range from zero for the case of axial symmetry to 1 in the fully rhombic limit. Spin Hamiltonian parameters of Y_*x*_Sc_3−*x*_N@C_80_ triplets listed in Table 2 were obtained from EPR spectra simulations using the EasySpin^96^ toolbox for Matlab.

**Table 2:** Spin Hamiltonian parameters of the Y_*x*_Sc_3−*x*_N@C_80_ excited triplet states

	*g* a		ZFS parameters^b^, MHz		Hyperfine coupling, MHz				Nuclear quadrupole coupling, MHz		
						^89^Y^c^	^45^Sc^d^	^14^N^c^		^45^Sc^e^	^14^N^f^
Y_3_N@C_80_	*g*_*x*_	2.0004	*D*	+128	*A*_⊥_	+1.02	—	+0.15	|*e*^2^*Qq*/*h*|	—	1.46
	*g*_*y*_	2.0008	*E*	0	*A*_∥_	+1.02	—	+0.62	*η*	—	0
	*g*_*z*_	2.0030									
Y_2_ScN@C_80_	*g*_*x*_	1.9964	*D*	+131	*A*_⊥_	−0.75	+35	+0.10	|*e*^2^*Qq*/*h*|	75	1.30
	*g*_*y*_	1.9984	*E*	0	*A*_∥_	−0.75	+49	−0.54	*η*	0	0
	*g*_*z*_	2.0025									
YSc_2_N@C_80_	*g*_*x*_	1.9986	*D*	+133	*A*_⊥_	—	+58	−0.64	|*e*^2^*Qq*/*h*|	75	1.21
	*g*_*y*_	1.9986	*E*	0	*A*_∥_	—	+58	−0.83	*η*	0	0
	*g*_*z*_	2.0003									
Sc_3_N@C_80_	*g*_*x*_	1.9969	*D*	+291	*A*_⊥_	—	+71	—	|*e*^2^*Qq*/*h*|	72	—
	*g*_*y*_	1.9969	*E*	0	*A*_∥_	—	+71	—	*η*	0	—
	*g*_*z*_	2.0002									

a The estimated error ±0.0002.

b The estimated error ±10 MHz for Sc-containing molecules because of Sc hyperfine broadening.

c The estimated error ±0.1 MHz.

d The estimated error ±3 MHz.

e The estimated error ±3 MHz.

f The estimated error ±0.05 MHz.

### Light-induced ENDOR

A quantitative description of the spin density distribution within the M_3_N clusters requires determination of hyperfine and quadrupolar couplings in eqn (2). As the EPR spectra do not provide sufficient resolution, the problem was addressed with Electron Nuclear Double Resonance spectroscopy (ENDOR). In pulsed ENDOR, the microwave electron-spin excitation pulse is followed by irradiation of the sample with a radiofrequency pulse, which changes the populations of nuclear spin levels and reduces the ESE intensity at the frequencies corresponding to the nuclear spin transitions. The frequencies of the ENDOR nuclear transitions, calculated to the first order of perturbation theory, are given by eqn (6):^97^6
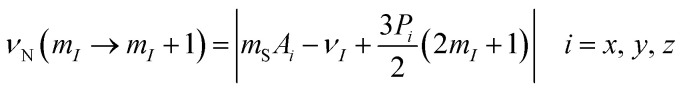
where *ν*_N_ represents the ENDOR line position, *m*_S_ and *m*_*I*_ the electron and nuclear magnetic quantum numbers, *A*_*i*_ the hyperfine coupling constant, *ν*_*I*_ is the Larmor frequency of the nuclear spin, and *P*_*i*_ is the corresponding quadrupole tensor principal value for nuclei with *I* > 1/2. In a weak coupling regime, |*ν*_*I*_| > |*A*_*i*_|, the ENDOR spectrum of the triplet state is centred around *ν*_*I*_ with peaks at *ν*_*I*_ and *ν*_*I*_ ± *A*_*i*_ or shows a more complex pattern for quadrupolar nuclei. Mims ENDOR is most suitable for studies of small hyperfine couplings, and this technique was applied in this work for the analysis of ^89^Y and ^14^N hyperfine interactions. In a strong coupling regime, |*ν*_*I*_| < |*A*_*i*_|, the spectrum of the triplet is centered around |*A*_*i*_| with peaks at |*A*_*i*_| ± *ν*_*I*_, and an additional feature near *ν*_*I*_, optionally split by quadrupolar interactions. Davies ENDOR is better suited for strong hyperfine couplings, and we used it for the studies of ^45^Sc hyperfine interactions.

### Mims ENDOR

**Fig. 11:**
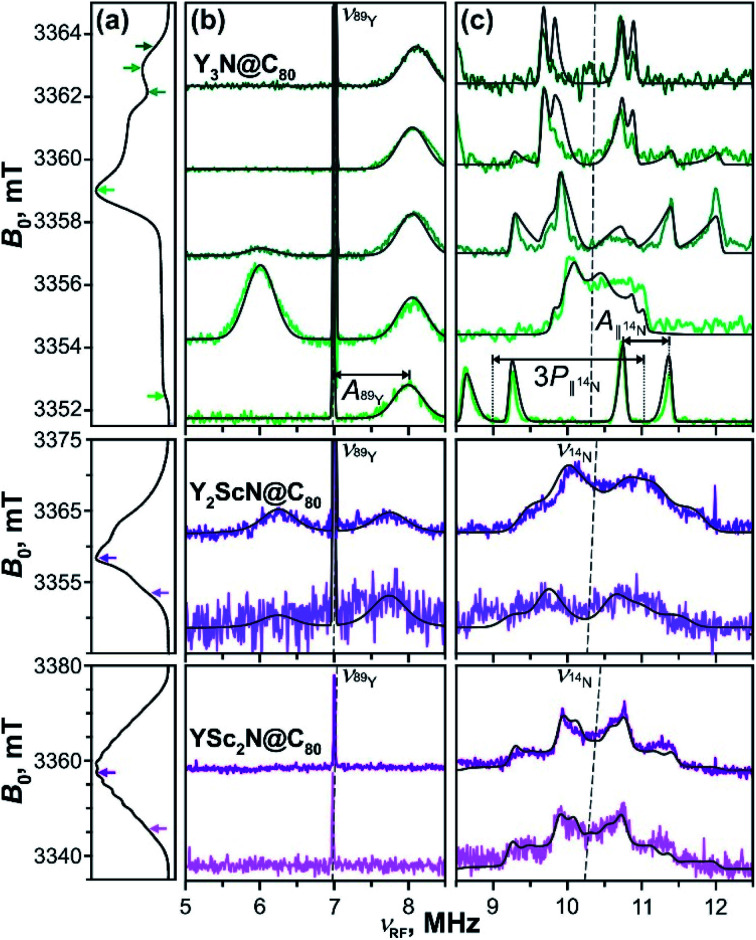
Mims-type (b) ^89^Y and (c) ^14^N ENDOR spectra of the Y_3_N@C_80_ (green lines), Y_2_ScN@C_80_ (violet lines) and YSc_2_N@C_80_ (purple lines) light-excited triplets recorded at magnetic field positions indicated by the arrows in panel (a) displaying the EPR spectra. The arrow colour tone matches with the corresponding experimental ENDOR trace. All spectra were obtained under continuous 510 nm irradiation at 20 K in frozen toluene-*d*_8_. Grey lines show the ENDOR spectra calculated using the spin Hamiltonian parameters summarized in Table 2. The splitting due to the corresponding hyperfine (*A*) and quadrupolar (*P*) interactions are illustratively indicated for Y_3_N@C_80_ in (c). The dashed lines indicate the Larmor frequencies of the corresponding nuclei.

Mims-type pulse W-band ENDOR data of the Y_*x*_Sc_3−*x*_N@C_80_ (*x* = 1–3) excited triplets are shown in Fig. 11. The spectra obtained with Y_3_N@C_80_ were already analyzed in our previous work^51^ and are reproduced here for completeness. The EPR spectrum of Y_3_N@C_80_ shows a well resolved ZFS fine structure and the ENDOR spectra were recorded at field positions corresponding to the turning points of the ZFS pattern, providing a sufficient orientation selection. Signals found at frequencies below 13.0 MHz reflect the hyperfine and quadrupole couplings of the cluster nuclei, ^89^Y and ^14^N.

Following eqn (6) for the *S* = 1 triplet state with *I* = 1/2 nuclei, the sharp line at 7 MHz (Fig. 11b, green) corresponds to the Larmor frequency of ^89^Y (*I* = 1/2, 100% natural abundance) and originates from the degenerate NMR transitions within the electron spin manifold *m*_S_ = 0. The two broader lines, spaced by *A*(^89^Y) from the *m*_S_ = 0 line, result from the ^89^Y hyperfine interaction at *m*_S_ = ±1. The invariant frequency position of these lines throughout the field-swept ENDOR series suggests the predominantly isotropic character of the ^89^Y hyperfine interaction, *A*_⊥_ ≈ *A*_∥_ ≈ *A*_iso_. Simulations of the spectra reveal a small positive isotropic hyperfine coupling (hfc) constant of *A*_iso_(^89^Y) = +1.02 MHz, with the three Y nuclei in the cluster being magnetically equivalent. In theory, the isotropic hyperfine coupling for the nucleus X is defined by the generalized Fermi contact term *A*_iso_(X) ∼ *S*^−1^*g*_n_(X)*ρ*_spin_(X),^98,99^ where *S* is the electron spin, *g*_n_(X) is the nuclear *g*-factor, and *ρ*_spin_(X) is the spin density at nucleus X. The factor *g*_n_(^89^Y) is negative, and the positive sign of the *A*_iso_(^89^Y) suggests that the electron spin density *ρ*_spin_(Y) at the Yttrium of the endohedral cluster is negative. This is in line with the results of our DFT calculations, which predict the positive sign for *A*_iso_(^89^Y) and thus a negative *ρ*_spin_(Y) generated by the spin polarization of ^89^Y s-orbitals (Table S7, ESI†).

The orientation selection obtained in the Mims ENDOR spectra of Y_3_N@C_80_ triplet also allows for a detailed analysis of the hyperfine and quadrupole tensor of the central cluster nitrogen (^14^N, *I* = 1, natural abundance 99.63%, Larmor frequency around 10.4 MHz, Fig. 11c, green lines). The simulation of experimental ENDOR recordings (Fig. 11c grey lines) reveals a rather small positive axial hfc with *A*_∥_(^14^N) = +0.62 MHz, *A*_⊥_(^14^N) = +0.15 MHz (*A*_iso_(^14^N) = +0.31 MHz), and a somewhat larger nuclear quadrupole of |*e*^2^*Qq*/*h*| = 1.46 MHz (asymmetry *η* = 0). The small positive *A*_iso_(^14^N) and the magnitude of the quadrupole coupling are also well predicted by DFT as detailed in Table S7.†

When compared to the Y_3_N@C_80_ data, the Mims ENDOR spectra of Y_2_ScN@C_80_ triplet (Fig. 11b) are of poorer quality. This is due to the shorter triplet lifetime, lower triplet quantum yield and a broader EPR spectrum resulting in a lower EPR intensity at the selected magnetic field position. Despite the lower signal-to-noise ratio, one can identify two ^89^Y hfc lines positioned closer to the central *m*_S_ = 0 line (Fig. 11b) suggesting a smaller *A*_iso_(^89^Y) of −0.75 MHz. While the claim on the isotropic character of the tensor and the equivalence of the two Y in the cluster is less justified, given the fewer field positions explored, we believe they hold. In this case, the negative sign of the *A*_iso_(^89^Y) is compatible with a small positive electron spin density *ρ*_spin_(Y) at Yttrium atoms. The negative sign of *A*_iso_(^89^Y) is also predicted by DFT (Table S7†), although the hfc values are overestimated by the calculation, and the two nuclei are predicted to be magnetically inequivalent. In contrast to Y_3_N@C_80_, the ^14^N ENDOR data (Fig. 11b) of Y_2_ScN@C_80_ show a stronger powder character. This is in line with the appearance of the EPR spectrum where the ZFS pattern, and thus the accessible orientation selection, smear out due to the hyperfine splitting from ^45^Sc. Simulations of the ENDOR data reveal an axial hyperfine tensor with *A*_∥_(^14^N) = −0.54 MHz, *A*_⊥_(^14^N) = +0.10 MHz (*A*_iso_(^14^N) = −0.11 MHz), and the nuclear quadrupole coupling of |*e*^2^*Qq*/*h*| = 1.30 MHz (asymmetry parameter *η* = 0) for the central nitrogen.

The experimental difficulties in obtaining high-quality ENDOR data are even more pronounced for YSc_2_N@C_80_, due to the physical limits given by the photophysical properties of its T_1_ state. We could not detect the *m*_S_ = ±1 ^89^Y hfc lines, even though the central *m*_S_ = 0 line is observable (Fig. 11b, purple). The ^14^N ENDOR data (Fig. 11b) show powder Pake patterns compatible with *A*_∥_(^14^N) = −0.83 MHz, *A*_⊥_(^14^N) = −0.64 MHz (*A*_iso_(^14^N) = −0.70 MHz), and the quadrupole coupling of |*e*^2^*Qq*/*h*| = 1.21 (asymmetry *η* = 0). The trend in the ^89^Y hyperfine couplings (Table 2), observed in the Y_*x*_Sc_3−*x*_N@C_80_ (*x* = 1–3) series, and particularly the switch of the sign of *A*_iso_(^89^Y) suggests an increase of the spin density on Y with increasing content of Sc in the cluster, supporting the idea of spin density transfer from the fullerene cage to the cluster. The interpretation of the differences in ^14^N couplings is less straightforward, but the DFT predicted small negative spin density *ρ*_spin_(N) at the nitrogen in Sc-containing molecules (negative *A*_iso_(^14^N), Table S7†) is reflected in the experimental data. In general, both ^89^Y and ^14^N hfc's are rather small. Therefore, we conclude that these are certainly not the spin-bearing nuclei in the T_1_ states of Y_*x*_Sc_3−*x*_N@C_80_ (*x* = 1–3).

### Davies ENDOR

**Fig. 12:**
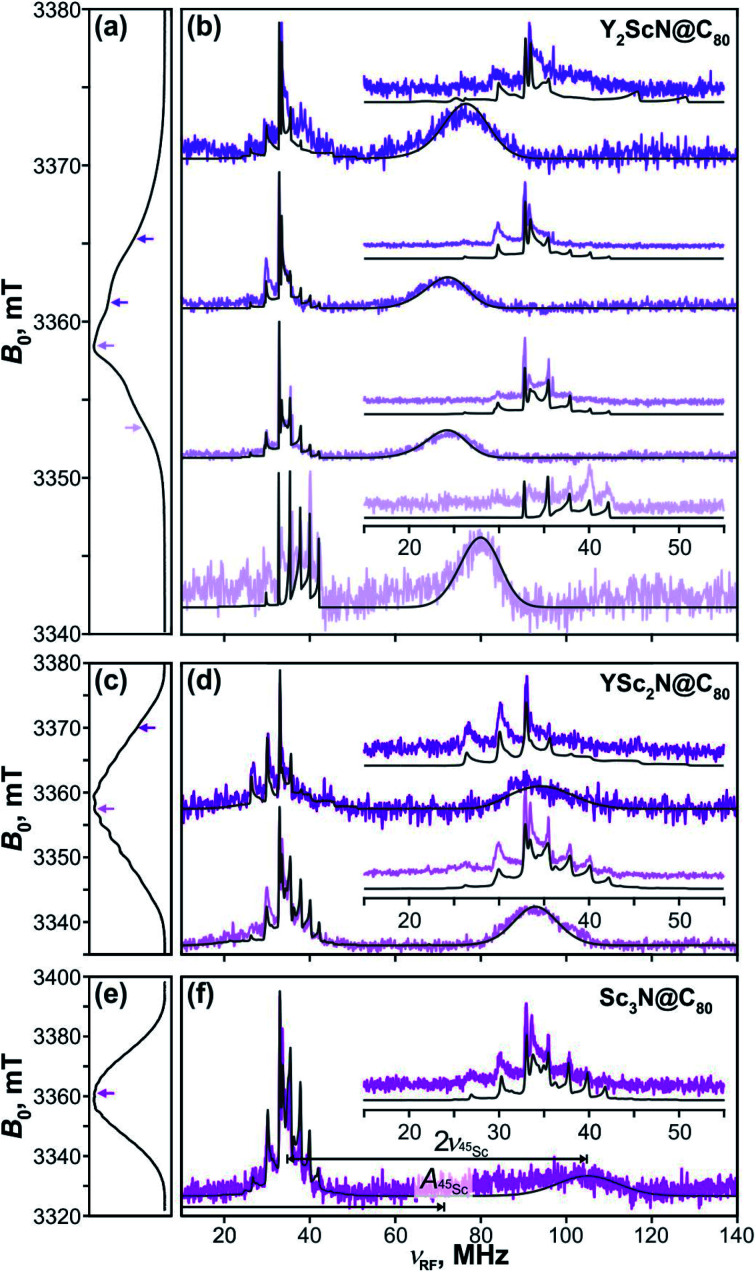
Davies-type ^45^Sc-ENDOR spectra (b) of Y_2_ScN@C_80_ (violet lines), (d) YSc_2_N@C_80_ (purple lines) and (f) Sc_3_N@C_80_ (magenta lines) excited triplet states detected at the arrow-marked magnetic field positions in W-band ESE EPR spectra in panels (a), (c), and (e), respectively. All spectra were obtained in frozen toluene-*d*_8_ at 20 K under continuous 510 nm laser irradiation. Insets in panels (b), (d), (f) show expansions of the signals around the ^45^Sc Larmor frequency, corresponding to the transitions within the *m*_S_ = 0 manifold. The well-resolved splitting patterns originate from the quadrupolar interaction. Grey lines show spectra calculated using the spin Hamiltonian parameters summarized in Table 2. The evaluation of the ^45^Sc hyperfine coupling 
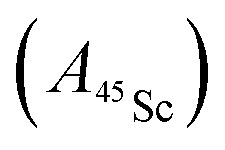
 is illustratively indicated for Sc_3_N@C_80_.

The key data to evaluate the hypothesis on the spin density transfer from cage to the Sc-containing cluster come from ^45^Sc ENDOR spectroscopy. The splittings in the EPR spectra of YSc_2_N@C_80_ and Sc_3_N@C_80_ triplets show that ^45^Sc hyperfine couplings are about 50 MHz. The ENDOR data for the Y_*x*_Sc_3−*x*_N@C_80_ (*x* = 0–2) triplets recorded at different magnetic field positions within the EPR spectra are shown in Fig. 12. To the best of our knowledge, these are the first ENDOR data reported for the ^45^Sc nucleus. In general, the spectra show well-resolved patterns at around 35 MHz and broad lines with linewidths exceeding 15 MHz above 60 MHz. With the ^45^Sc Larmor frequency around 34.8 MHz, the obtained data correspond to ENDOR spectroscopy in the high coupling regime with 
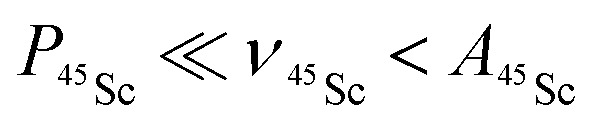
. In this regime, according to eqn (6), the spectrum of the *S* = 1 triplet is expected to feature a doublet signal centered at the frequency of the hyperfine coupling and split by double the Larmor frequency, as indicated in Fig. 12f (magenta, bottom panel). Additionally, the nuclear transitions within the *m*_S_ = 0 manifold generate a signal at the ^45^Sc nuclear Larmor frequency, that is influenced by nuclear quadrupole coupling and higher-order hyperfine shifts.

The ^45^Sc ENDOR spectra of Y_2_ScN@C_80_ (Fig. 12b, violet lines) reveal a shift of the position of the high-frequency line (*m*_S_ = −1) from 65 to 80 MHz, depending on the field position within the EPR spectrum, indicating a minor anisotropy of the ^45^Sc hyperfine coupling. The line broadening is likely the consequence of a complex quadrupolar splitting combined with a hyperfine strain. The signal is readily detectable, despite the broadening, due to the hyperfine-enhancement effect^100,101^ producing larger ENDOR intensity for the high-frequency component of the hyperfine doublet. The low-frequency counterpart (*m*_S_ = +1) essentially vanishes in the noise at the low-frequency end of the ENDOR spectrum and partially overlaps with the well-resolved *m*_S_ = 0 feature at the Larmor frequency. The spectral simulations revealed that an axial hyperfine tensor with *A*_∥_(^45^Sc) = 49 MHz and *A*_⊥_(^45^Sc) = 35 MHz is compatible with the field-dependent shifts of the high-frequency line. The well resolved Pake patterns at the ^45^Sc Larmor frequency reflect an axial quadrupolar coupling tensor of the high-spin *I* = 7/2 ^45^Sc nucleus defined by |*e*^2^*Qq*/*h*| = 75 MHz and asymmetry *η* = 0 (Fig. 12b, violet lines).

The ^45^Sc ENDOR spectra of YSc_2_N@C_80_ show less pronounced variations of the position of the high-frequency line and the spectra calculated with isotropic hyperfine tensors for both Sc atoms, *A*_iso_(^45^Sc) = *A*_∥_(^45^Sc) = *A*_⊥_(^45^Sc) = 58 MHz, match reasonably well with the experimental traces (Fig. 12d, purple lines). The isotropic character of the coupling is also in line with the well-resolved and regular splitting observed in the corresponding EPR spectrum (Fig. 10). The quadrupolar coupling extracted from the sharp peaks around the Larmor frequency is essentially identical with the Y_2_ScN cluster |*e*^2^*Qq*/*h*| = 75 MHz (*η* = 0).

The ENDOR spectrum of Sc_3_N@C_80_ triplet (Fig. 12f, magenta line) recorded at the field position of the maximum EPR signal shows the high-frequency line at 104 MHz and suggest a hyperfine tensor with *A*_iso_(^45^Sc) = *A*_∥_(^45^Sc) = *A*_⊥_(^45^Sc) = 71 MHz. Since here we have the data only from a single magnetic field position, the isotropic character of the tensor is an estimate based on the well-resolved hyperfine pattern observed in the EPR (Fig. 10). The low-frequency hyperfine ENDOR line (*m*_S_ = +1) is in this case completely covered by the *m*_S_ = 0 multiplet, which can be reproduced in the calculated spectrum with the quadrupolar coupling of |*e*^2^*Qq*/*h*| = 72 MHz (*η* = 0). Note that the *A*_iso_(^45^Sc) in the triplet state is about twice smaller than in the Sc_3_N@C_80_^−^ anion with *A*_iso_(^45^Sc) ≈ 150 MHz.^90–93^ The DFT-predicted spin density and spin population at Sc in both species are almost identical, but the *S*^−1^ scaling of the hfc constant leads to a two-fold decrease of the *A*_iso_(^45^Sc) value for the triplet (*S* = 1) in comparison to the anion radical (*S* = 1/2). Alternatively, it can be recalled that the hfc constant in the triplet state is expected to be close to an average of the hfc constants of the anion and cation radicals (see ref. 102 for an example). As the HOMO in Sc_3_N@C_80_ is localized on the fullerene cage, the ^45^Sc hfc constant of the cation should be very small (experimental value is not known yet), which gives *A*_iso_(^45^Sc, T_1_) ≈ 0.5*A*_iso_(^45^Sc, anion).

Summarizing, the ^45^Sc ENDOR of the Y_*x*_Sc_3−*x*_N@C_80_ (*x* = 0–2) reveals an increase in the magnitude of the ^45^Sc hyperfine coupling with the increasing number of Sc atoms in the cluster, in line with the predicted fullerene-to-metal spin density transfer in the photoexcited triplets. The ENDOR spectroscopy of triplet *S* = 1 states additionally provides a unique method to obtain detailed information about the nuclear quadrupolar interaction, since the corresponding splittings are well resolved with the *m*_S_ = 0 multiplet. The ^45^Sc quadrupolar coupling in Y_*x*_Sc_3−*x*_N@C_80_ (*x* = 0–2) excited triplet series is essentially identical and is very close to the value for the parent ground state of Sc_3_N@C_80_ revealed by solid state-NMR (|*e*^2^*Qq*/*h*| = 67.9 MHz, *η* = 0).^103^ This similarity suggests that the electric field gradients around the Sc nuclei are of similar magnitude and are not dramatically influenced by the electron and spin density redistribution in the S_0_ → T_1_ excitation.

Utilizing the detailed data on the hyperfine and quadrupolar tensors (Table 2) obtained from the ENDOR investigation we were able to calculate the EPR spectra of all the Y_*x*_Sc_3−*x*_N@C_80_ (*x* = 0–3) triplets shown in Fig. 10 (grey lines). The calculated EPR spectra match well with the experimental recordings, when we assume that the Sc atoms in the YSc_2_N and Sc_3_N clusters are magnetically equivalent. The zero-field splitting tensors were considered axially symmetric and the *D* values grow from +128 MHz in Y_3_N@C_80_ to +291 MHz in Sc_3_N@C_80_. The *D* value appears similar for Y_3_N@C_80_, YSc_2_N@C_80_, and Y_2_Sc@NC_80_, but precise details cannot be discussed because of the uncertainty in the value introduced by the broadening of the spectra by Sc-hyperfine interaction. Additionally, a decrease in all principal values of the *g*-tensor is observed with increasing content of Sc in the cluster. Together with the increase of the ^45^Sc hyperfine coupling throughout the series, these data confirm the progressive shift of the spin density from the fullerene cage to the Sc-containing cluster and are fully consistent with the conclusions from the photochemical investigations and the DFT-modelling. Hyperfine and quadrupolar parameters and *g*-tensors of the lowest-energy triplet conformers were also computed with DFT and listed in Table S7.† The computations qualitatively reproduce the experimental trends, including a decrease of the g-factor from Y_3_N@C_80_ to Y_2_ScN@C_80_ to YSc_2_N@C_80_ as well as the increase of the *A*(^45^Sc) hyperfine constants. DFT calculations also point out that the *A*(^45^Sc)-tensors do not show a large anisotropy, thus justifying an isotropic approximation employed in simulations of the experimental EPR and ENDOR spectra. Finally, the DFT calculated quadrupolar parameters are also in close agreement with the experimental values. The predicted asymmetry parameter *η* is close to 0 in all studied structures for both ^14^N and ^45^Sc, which agrees with experimental observations.

## Conclusions

A systematic study of the photophysical properties of metallofullerenes Y_*x*_Sc_3−*x*_N@C_80_ (*x* = 0–3) showed that the cluster composition has a very strong influence on the emission properties of these, seemingly similar nitride clusterfullerene. Utilizing variable-temperature steady-state and time-resolved measurements we proved that luminescence from all four compounds follows the thermally-activated delayed fluorescence mechanism, replaced by phosphorescence only at very low temperature. All Y_*x*_Sc_3−*x*_N@C_80_ molecules feature a very small S_1_–T_1_ gap of less than 0.1 eV, which indicates that TADF may be a universal phenomenon in metallofullerenes. Emission studies also revealed a strong increase of the luminescence wavelength with the increase of the number of Sc atoms in the endohedral cluster, which is not reflected in the absorption spectra. Such changes point to a potential structural reorganization upon excitation, and this hypothesis was thoroughly analyzed by theoretical modelling of the ground and excited states. Indeed, we found that for all M_3_N clusters with Sc atoms, the M_3_N cluster has different preferential positions inside the fullerene cage in the S_0_ and S_1_/T_1_ states. Furthermore, computations revealed that the conformers with lower energy in the excited state tend to increase the localization of the excess electron charge and spin density on Sc atoms compared to the conformers preferred in the ground state. Light-induced pulsed W-band EPR spectroscopy was applied to further corroborate the properties of the triplet states. The triplet lifetimes found by electron spin echo detected EPR are in good agreement with optical measurements, thus confirming the correct assignment of the emitting states. Mims and Davies-type ^14^N, ^45^Sc, and ^89^Y ENDOR measurements provided a detailed characterization of the spin distribution in the triplet states and showed a systematic increase of the spin density localization on Sc atoms when going from Y_2_ScN@C_80_ to Sc_3_N@C_80_, in perfect agreement with computational results. Altogether, our study demonstrates that the position of the endohedral cluster in metallofullerenes can be controlled by light excitation, and that the driving force for the photoswitching is the fullerene-to-cluster charge transfer and localization of the electron charge and spin density on the metal atoms.

The flexibility of the metal cluster in EMFs often results in disorder of the metal positions, which substantially affects the studies of their molecular structure and physical properties, such as electron transport or magnetism. Whereas conventional cooling of metallofullerene assemblies usually results in disordered cluster positions, light-induced switching can be used for better control of such assemblies. For instance, light-assisted annealing can be developed to selectively increase the population of particular cluster orientations, whereas a combination of this effect with lanthanide-based magnetism in metallofullerenes^104,105^ opens a way to magnetooptical switching on a nanoscale.

## Author contributions

F. Z. and S. S. performed luminescence studies; V. D., S. M. A. and A. A. P. performed DFT calculations; S. H. D. and X. B. W. performed the PES study; M. Z., A. S. and W. L. performed EPR study; M. Z. and A. A. P. conceived the study and written the manuscript with contributions from all coauthors.

## Conflicts of interest

There are no conflicts to declare.

## Supplementary Material

SC-012-D0SC07045A-s001
