# Supplementary material for: Metallofullerene photoswitches driven by photoinduced fullerene-to-metal electron transfer
Source: Chem Sci. 2021 Apr 30;12(22):7818–38. doi: 10.1039/d0sc07045a (PMC8188499; doi:10.1039/d0sc07045a)
Supplement: SC-012-D0SC07045A-s001 [file SC-012-D0SC07045A-s001.pdf]

Electronic Supplementary Material (ESI) for Chemical Science.
This journal is © The Royal Society of Chemistry 2021

Metallofullerene photoswitches driven by photoinduced fullerene-to-metal electron transfer

Michal Zalibera,[a,b]* Frank Ziegs,[c] Sandra Schiemenz,[c] Vasilii Dubrovin,[c] Wolfgang Lubitz,[b] Anton Savitsky,[b,d] Shihu H. M. Deng,[e] Xue-Bin Wang,[e] Stanislav M. Avdoshenko,[c] Alexey A. Popov[c]*

Table of contents

Photophysical measurements S2

DFT and TD-DFT calculations S7

PES measurements S17

EPR measurements S18

References S20

Samples

Nitride clusterfullerenes $Y_xSc_{3-x}N@C_{80}$ (x = 0–3) were synthesized in our group earlier, using the protocol similar to the described in ref. [1], but employing NH_3 as a source of nitrogen.[2]

Photophysical studies

Absorption spectra were measured with Shimadzu dispersive spectrophotometer.

Steady-state emission spectra were excited with Omicron PhoxX diode lasers (405 and 488 nm lines) and detected with Avantes AvaSpec HS1024x122TEC high-sensitivity fiber-optic spectrometer with TE-cooled backthinned CCD detector. To get over the detector limits of Si-based CCDs for $Sc_3N@C_{80}$, AvaSpec NIR 256 1.7 spectrometer (Avantes) was used. The measurement in the NIR range beyond 1000 nm did not reveal new features in the luminescence spectra of $Sc_3N@C_{80}$.

Variable temperature measurements of fullerenes dispersed in polystyrene film were performed with fluorescence microscope of local design and Janis ST-500 microscopy cryostat (temperatures down to 4 K) or an Oxford Instruments MO2 cryostat. At temperatures below liquid nitrogen, a certain temperature gradient evolves inside a contact cryostat between the cold finger and a sample. Besides, the laser irradiation also results in local heating of the sample (the effect is however diminished in time-resolved measurements with long waiting time between laser pulses). These effects may lead to a temperature difference between a sample and the cold finger up to 5–8 K. The temperature values listed in Table S2-S4 and the main text are recordings of the controller, and the real temperature at the sample may be somewhat higher.

Additional variable-temperature measurements were performed for $Y_3N@C_{80}$ in degassed toluene solution. The sampled was sealed in the glass tubed and cooled using a nitrogen-flow cryostate of home design.

Luminescence lifetimes were measured by time-correlated single-photon counting (TCSPC) method. Luminescence was excited by a digitally modulated Omicron PhoxX diode laser, time-resolved detection was performed by a PMT PMA 192 (PicoQuant) or an ID230 NIR single-photon counter (ID Quantique) and acquired by a time-correlated single-photon counting system (TCSPC) based on TimeHarp 260 counter/timer (PicoQuant) and the FluoFit software. When emission was intense enough, the emitted light was passed through a 9030 compact monochromator (Sciencetech) (≈20 nm maximal bandwidth) to the detector. For week emission intensities, a broadband regime was used either with long-pass (> 650 nm) or band-pass filters (820–880 nm). The ID230 detector and the scanning monochromator together with the SciSpec software was used to record continuous and time-resolved luminescence of $Sc_3N@C_{80}$ at wavelengths longer than 900 nm. Note that chosen detection range not necessarily corresponds to the maximum PL intensity but was chosen for optimal measurement in a broader temperature range.

Examples of decay curves measured for $Y_3N@C_{80}$ are shown in Fig. S1. Tables S1–S4 list determined luminescence lifetimes together with the detection range.

Photoluminescence decays curves

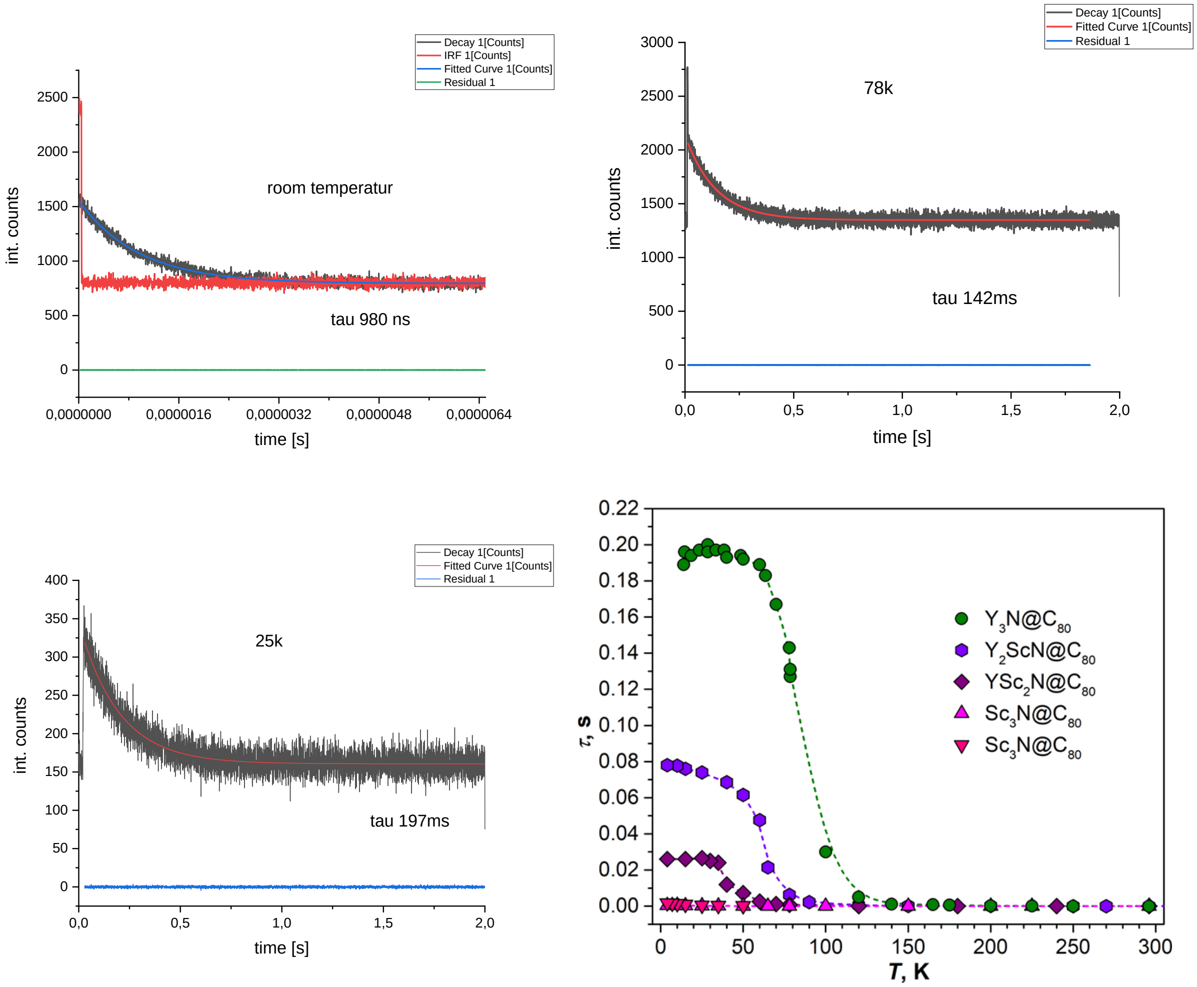

Figure S1. Selected PL decays curves recorded for $Y_3N@C_{80}$ at room temperature, 78 K, and 25 K, and their fits with exponential decay, and residual. Right bottom corber shows PL lifetimes in linear scale.

Table S1. Luminescence lifetimes of $Y_3N@C_{80}$ in polystyrene films and edgassed toluene solution measured at different temperatures

T, K	*τ*, ms, polymer	*τ*, ms, toluene	detection
14	189		monochromator @740 nm
14.5	196		monochromator @740 nm
18.5	194		monochromator @740 nm
23.5	197		monochromator @740 nm
28.5	200		monochromator @740 nm
28.5	196		monochromator @740 nm
33.5	197		monochromator @740 nm
38.5	197		monochromator @740 nm
40	193		monochromator @740 nm
48.5	194		monochromator @740 nm
50	192		monochromator @740 nm
60	189		monochromator @740 nm
63.5	183		monochromator @740 nm
70	167		monochromator @740 nm
78	143		monochromator @740 nm
78.5	127		monochromator @740 nm
78.5	131		monochromator @740 nm
100	30		monochromator @701 nm
120	5		monochromator @701 nm
140	1.05		monochromator @701 nm
165	0.878	0.620	monochromator @706 nm
175	0.528	0.405	monochromator @706 nm
200	0.165	0.0954	monochromator @706 nm
225	0.0265	0.0247	monochromator @706 nm
250	0.0072	0.0065	monochromator @706 nm
296	0.00108	0.00095	monochromator @706 nm
325	0.00045		monochromator @706 nm
335	0.00035		monochromator @706 nm

Table S2. Luminescence lifetimes of $Y_2ScN@C_{80}$ in polystyrene films measured at different temperatures

T, K	*τ*, ms	detection
4	78	Filter long-pass 650 nm
10	77.7	Filter long-pass 650 nm
15	76	Filter long-pass 650 nm
25	74	Filter long-pass 650 nm
40	68.6	Filter long-pass 650 nm
50	61.5	Filter long-pass 650 nm
60	47.6	Filter long-pass 650 nm
65	21.5	monochromator @770 nm
78	6.27	monochromator @753 nm
90	2.17	monochromator @753 nm
120	0.429	monochromator @753 nm
150	0.125	monochromator @753 nm
200	0.044	monochromator @753 nm
250	0.018	monochromator @753 nm
270	0.015	monochromator @753 nm
296	0.0105	monochromator @750 nm
396	0.003	monochromator @740 nm

Table S3. Luminescence lifetimes of $YSc_2N@C_{80}$ in polystyrene films measured at different temperatures

T, K	*τ*, ms	detection
4	26	Filter long-pass 650 nm
15	26	Filter long-pass 650 nm
25	26.5	Filter long-pass 650 nm
30	25	Filter long-pass 650 nm
35	24	Filter long-pass 650 nm
40	12	Filter long-pass 650 nm
50	7.2	Filter long-pass 650 nm
60	2.5	monochromator @800 nm
70	1.1	monochromator @800 nm
78	0.77	monochromator @800 nm
120	0.049	monochromator @800 nm
180	0.0068	monochromator @800 nm
240	0.0022	monochromator @800 nm
296	0.0012	monochromator @800 nm

Table S4. Luminescence lifetimes of $Sc_3N@C_{80}$ in polystyrene films measured at different temperatures

T, K	τ, µs	τ, µs	τ, µs	detection[a]
4	400	1800	0.0014	band-pass 820–880 nm
7	400	1640		band-pass 820–880 nm
10	410	1360		band-pass 820–880 nm
15	240	1000		band-pass 820–880 nm
25	160	510		band-pass 820–880 nm
35	120	340	0.0017	band-pass 820–880 nm
50	64	250		band-pass 820–880 nm
65	13			band-pass 820–880 nm
78	5			band-pass 820–880 nm
100	2.55		0.0028	band-pass 820–880 nm
150	0.66		0.0070	band-pass 820–880 nm
200	0.24		0.0088	band-pass 820–880 nm
225	0.13			band-pass 820–880 nm
296	0.3			band-pass 820–880 nm

[a] In addition to the measureemmnts in the 820-880 nm range with PMT PMA 192, test PL lifetime measureemnt of $Sc_3N@C_{80}$ were also performed at λ>1000 nm with ID230 NIR single photon counter. These measurements did not reveal processes with different lifetimes and are thus not discussed.

DFT calculations

Computational details. Each structure was optimized in the S_0 and T_1 electronic states at the PBE level using the Priroda code[3, 4] with the implemented basis set of TZ2P quality with an effective core potential for Sc and Y atoms. Each unique conformer found in this screening was verified to be a true minimum by a Hessian calculation. Optimized coordinates of unique T_1 conformers were then re-optimized in the S_1 state at 5the TD-DFT level. Single point energy calculations at the PBE/def2-TZVPP level with ZORA scalar-relativistic corrections were then performed for all unique conformers in S_0, S_1, and T_1 states using ORCA suite, which was also used for calculations of hyperfine tensors and g-factors in the triplet state.[5-11]

Relative energies of all computed conformers in S_0, T_1, and S_1 states are listed in Table S5, whereas Figures S2-S3 show isosurfaces of HOMO, LUMO, difference density $\Delta\rho(S_0 \rightarrow S_1)$, and spin density $\rho_{spin}(T_1)$ for selected conformers

Table S5. Relative energies of $Y_xSc_{3-x}N@C_{80}$ conformers in S_0, S_1, and T_1 electronic states (in eV)

		S_0	$S_0\{S_1\}$	$S_0\{T_1\}$	S_1	$S_1\{S_0\}$	T_1	$T_1\{S_0\}$	Δ_{ST}	$\rho_{spin}(T_1)$
$Y_3N@C_{80}$	**conf 1**	0.000	0.067	0.068	1.554	1.599	1.451	1.498	0.104	0.08
	**conf 2**	0.062	0.144	0.131	1.568	1.649	1.494	1.569	0.073	0.17
	**conf 3**	0.069	0.150	0.138	1.568	1.665	1.492	1.583	0.076	0.18
$Y_2ScN@C_{80}$	**conf 1**	0.000	0.082	0.077	1.516	1.576	1.424	1.483	0.092	0.15
	**conf 2**	0.044	0.145	0.131	1.463	1.548	1.389	1.477	0.074	0.28
	**conf 3**		0.138	0.128	1.438		1.375		0.063	0.30
	**conf 4**		0.299	0.245	1.553		1.471		0.082	0.23
	**conf 5**		0.224	0.224	1.466		1.445		0.021	0.41
$YSc_2N@C_{80}$	**conf 1**	0.000				1.564		1.488		
	**conf 2**	0.001				1.544		1.458		
	**conf 3**	0.012	0.111	0.100	1.312	1.436	1.249	1.366	0.062	0.42
	**conf 4**	0.028	0.080	0.075	1.444	1.489	1.359	1.401	0.084	0.23
	**conf 5**	0.041	0.120	0.118	1.350	1.443	1.296	1.379	0.054	0.43
	**conf 6**		0.320	0.284	1.408		1.335		0.073	0.43
	**conf 7**		0.329	0.263	1.426		1.359		0.067	0.35
	**conf 8**		0.193	0.185	1.336		1.302		0.034	0.56
	**conf 9**		0.350	0.227	1.445		1.422		0.022	0.38
$Sc_3N@C_{80}$	**conf 1**	0.000	0.133	0.081	1.430	1.503	1.356	1.421	0.074	0.29
	**conf 2**	0.024	0.127	0.111	1.239	1.357	1.195	1.302	0.044	0.56
	**conf 3**	0.068	0.161	0.151	1.178	1.267	1.148	1.234	0.030	0.67
	**conf 4**	0.071				1.501		1.425		0.58
	**conf 5**		0.305	0.287	1.264		1.238		0.025	0.41
	**conf 6**		0.227	0.188	1.381		1.324		0.056	0.35
	**conf 7**		0.187	0.174	1.430		1.368		0.062	0.46
	**conf 8**		0.361	0.334	1.443		1.380		0.063	0.29

Relative energies are referred to the lowest-energy conformers of the ground electronic state; "S_0" denotes the energy of the S_0 state in optimized S_0 geometry, "$S_0\{S_1\}$" is the energy of the S_0 state in the optimized S_1 geometry, and "$S_0\{T_1\}$" is the energy of the S_0 state in the optimized T_1 geometry; Δ_{ST} is the adiabatic energy difference between S_1 and T_1, and $\rho_{spin}(T_1)$ is net Mulliken spin population of the M_3N cluster in the triplet state

Table S6. Relative adiabatic energies of $Sc_3N@C_{80}$ conformers in T_1 and monoanionic state (in eV)

	T_1, Δ-SCF		*T_1,* TD-DFT		$Sc_3N@C_{80}^-$	
	ΔE vs **conf 1**, *S_0*	*ΔE* vs **conf 3**, *T_1*	*ΔE* vs **conf 1**, *S_0*	*ΔE* vs **conf 3**, *T_1*	*EA* vs **conf 1**, *S_0*	*ΔE* vs **conf 3**, *anion*
**conf 3**	1.220	0.000	1.148	0.000	2.683	0.000
**conf 2**	1.245	0.025	1.195	0.046	2.660	0.023
**conf 5**	1.313	0.092	1.238	0.090	n/a	n/a
**conf 6**	1.384	0.164	1.324	0.176	2.495	0.187
**conf 1**	1.386	0.166	1.356	0.208	2.500	0.183
**conf 7**	1.405	0.185	1.368	0.220	2.480	0.203
**conf 8**	1.433	0.213	1.380	0.231	2.459	0.224

Δ-SCF denotes computations of the triplet state by ground-state DFT with spin multiplicity $2S+1$ = 3. TD-DFT denotes computations of the triplet state energy with TD-DFT, using geometry optimized with *Δ*-SCF approach. The energies are referenced versus **conf 1** in its *S_0* state (which gives excitation energies), or versus **conf 3** in its T_1 state (which gives relative energies of conformers). For the anions, electron affinity *EA* is computed as the energy difference of the optimized anion of a given conformer and the neutral $Sc_3N@C_{80}$, **conf 1** in S_0 state.

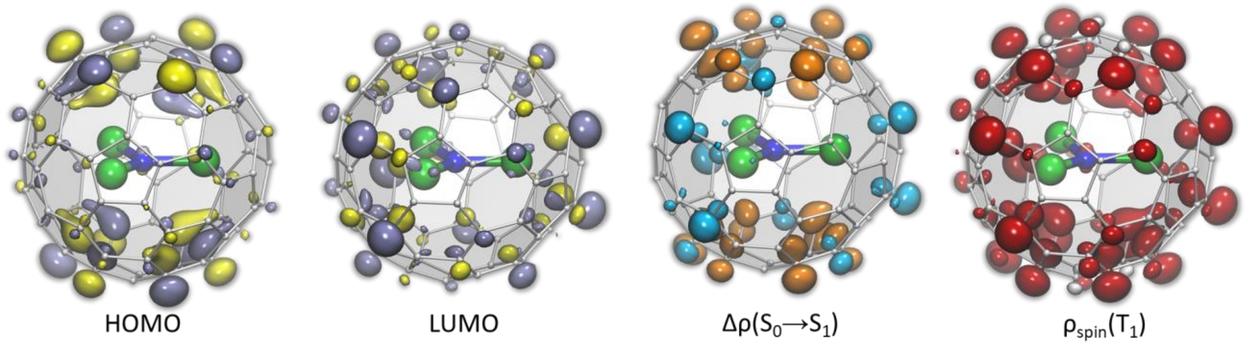

Figure S2a. Isosurfaces of HOMO, LUMO, difference density $\Delta\rho(S_0 \rightarrow S_1)$, and spin density $\rho_{spin}(T_1)$ computed for $Y_3N@C_{80}$, **conf 1**

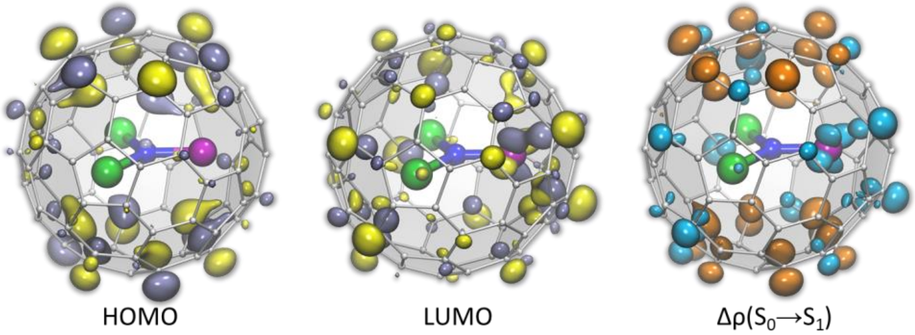

Figure S2b. Isosurfaces of HOMO, LUMO, and difference density $\Delta\rho(S_0 \rightarrow S_1)$ computed for $Y_2ScN@C_{80}$, **conf 1**

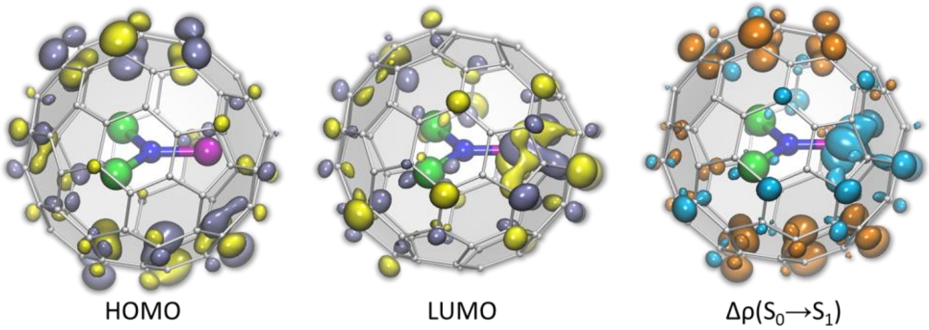

Figure S2c. Isosurfaces of HOMO, LUMO, and difference density $\Delta\rho(S_0 \rightarrow S_1)$ computed for $Y_2ScN@C_{80}$, **conf 2**

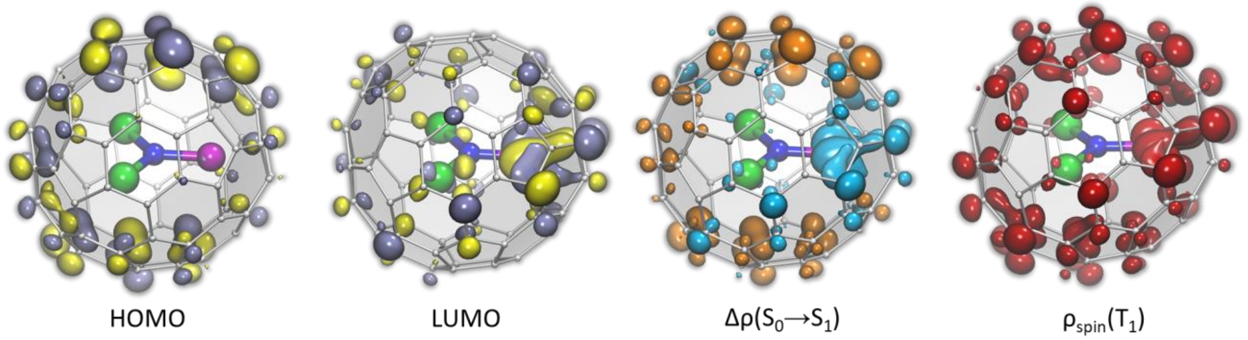

Figure S2d. Isosurfaces of HOMO, LUMO, difference density $\Delta\rho(S_0\rightarrow S_1)$, and spin density $\rho_{spin}(T_1)$ computed for $Y_2ScN@C_{80}$, **conf 3**

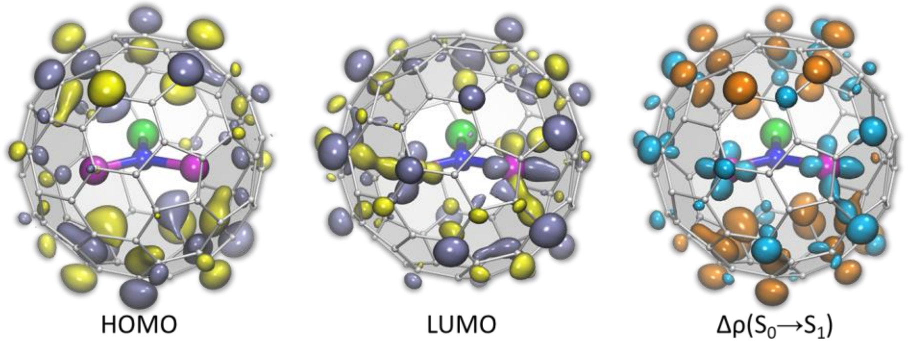

Figure S2e. Isosurfaces of HOMO, LUMO, and difference density $\Delta\rho(S_0\rightarrow S_1)$ computed for $YSc_2N@C_{80}$, **conf 1**

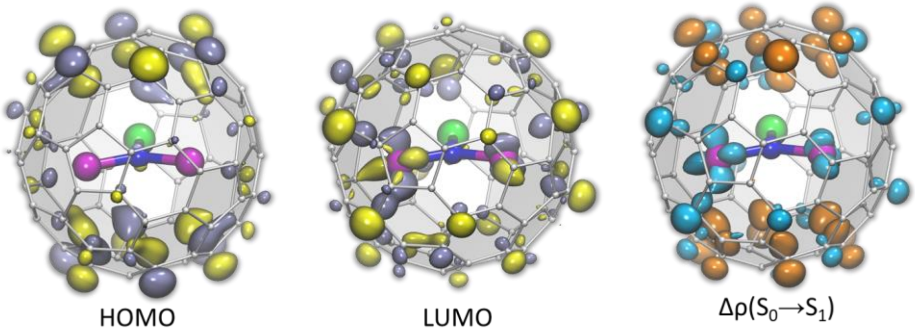

Figure S2f. Isosurfaces of HOMO, LUMO, and difference density $\Delta\rho(S_0\rightarrow S_1)$ computed for $YSc_2N@C_{80}$, **conf 2**

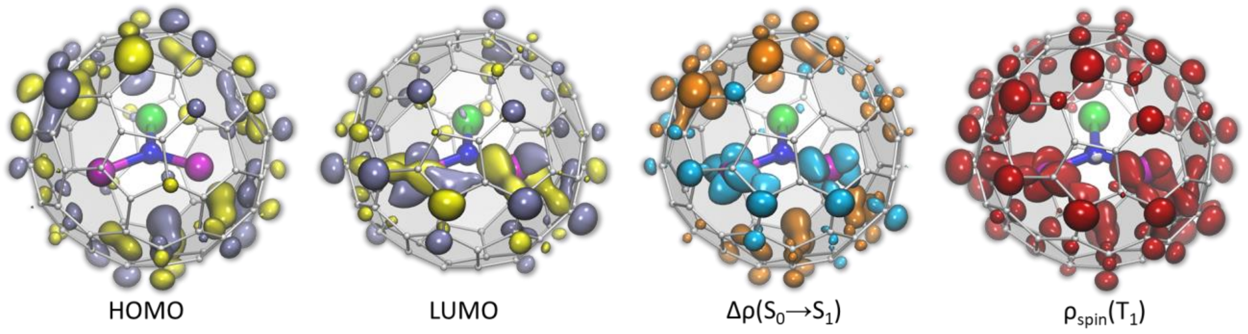

Figure S2g. Isosurfaces of HOMO, LUMO, difference density $\Delta\rho(S_0 \rightarrow S_1)$, and spin density $\rho_{spin}(T_1)$ computed for $YSc_2N@C_{80}$, **conf 3**

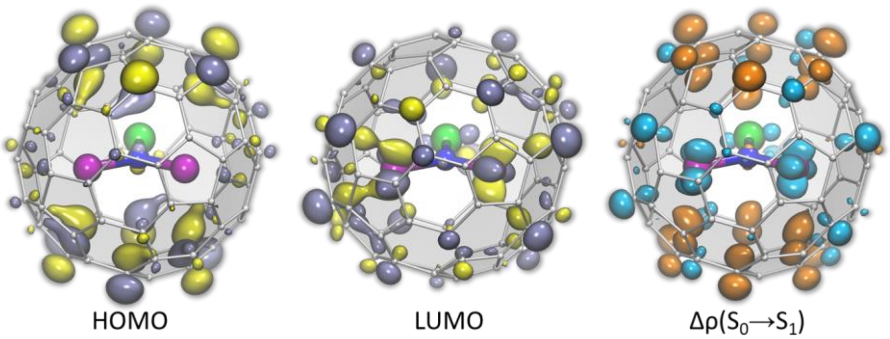

Figure S2h. Isosurfaces of HOMO, LUMO, and difference density $\Delta\rho(S_0 \rightarrow S_1)$ computed for $YSc_2N@C_{80}$, **conf 4**

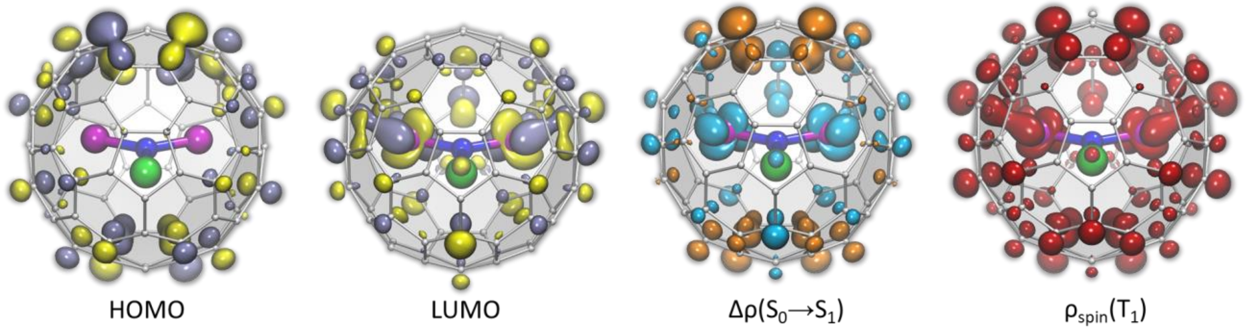

Figure S2i. Isosurfaces of HOMO, LUMO, difference density $\Delta\rho(S_0 \rightarrow S_1)$, and spin density $\rho_{spin}(T_1)$ computed for $YSc_2N@C_{80}$, **conf 5**

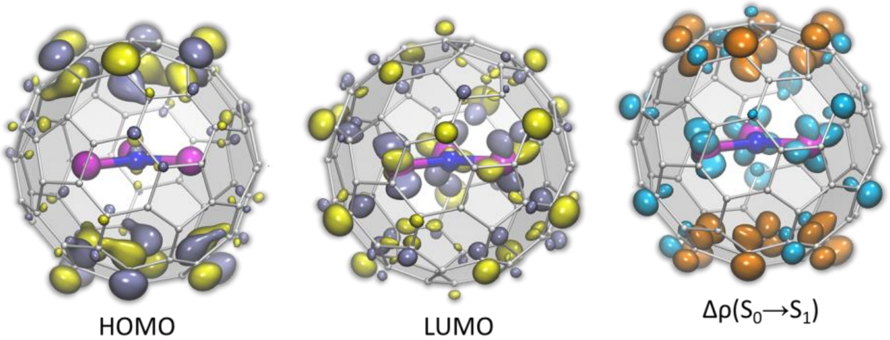

Figure S2j. Isosurfaces of HOMO, LUMO, and difference density $\Delta\rho(S_0\rightarrow S_1)$ computed for $Sc_3N@C_{80}$, **conf 1**

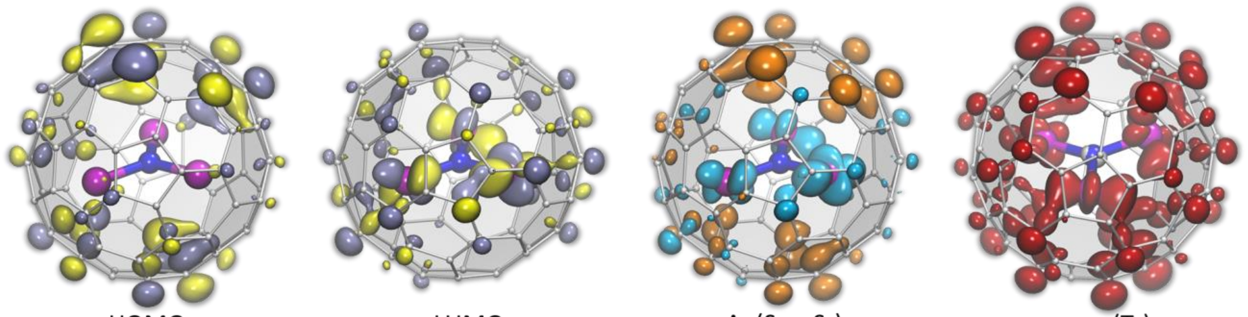

Figure S2k. Isosurfaces of HOMO, LUMO, difference density $\Delta\rho(S_0\rightarrow S_1)$, and spin density $\rho_{spin}(T_1)$ computed for $Sc_3N@C_{80}$, **conf 2**

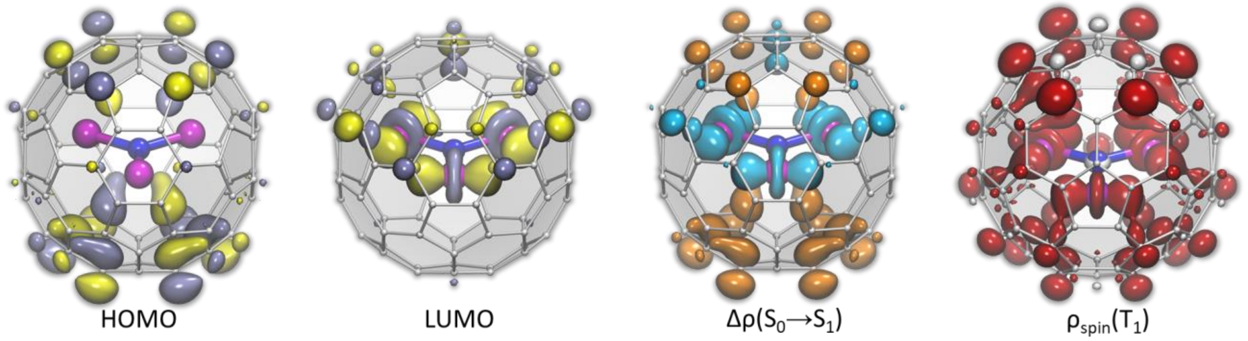

Figure S2l. Isosurfaces of HOMO, LUMO, difference density $\Delta\rho(S_0\rightarrow S_1)$, and spin density $\rho_{spin}(T_1)$ computed for $Sc_3N@C_{80}$, **conf 3**

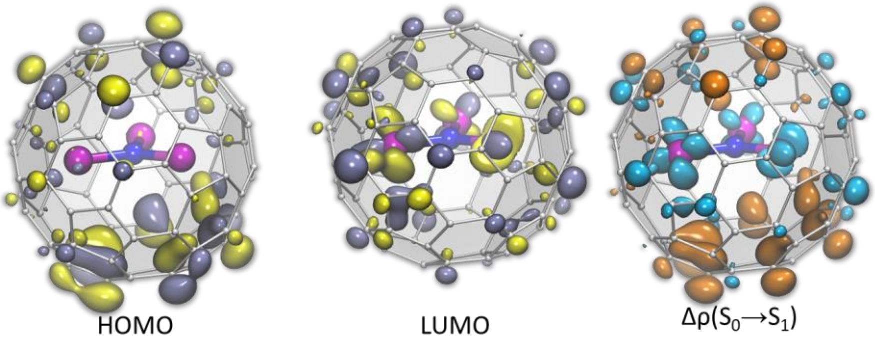

Figure S2m. Isosurfaces of HOMO, LUMO, and difference density $\Delta\rho(S_0 \rightarrow S_1)$ computed for $Sc_3N@C_{80}$, **conf 4**

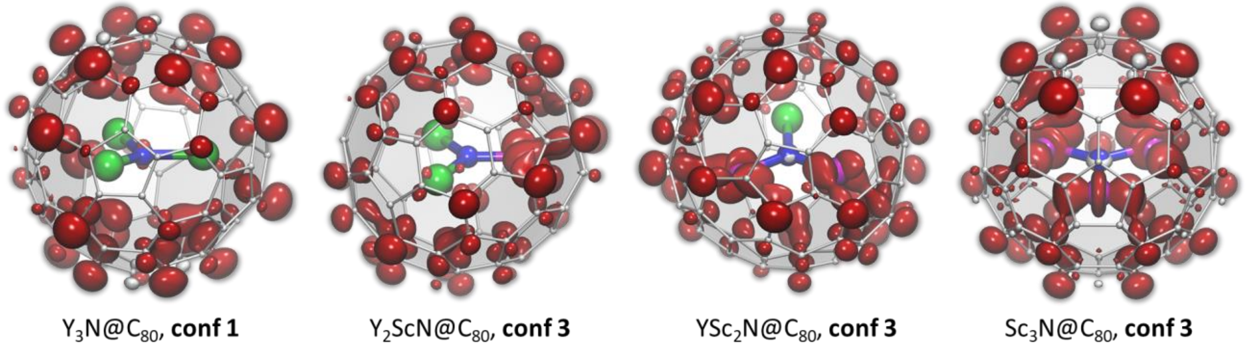

Figure S3. Isosurfaces of spin density $\rho_{spin}(T_1)$ of the lowest-energy T_1 conformers

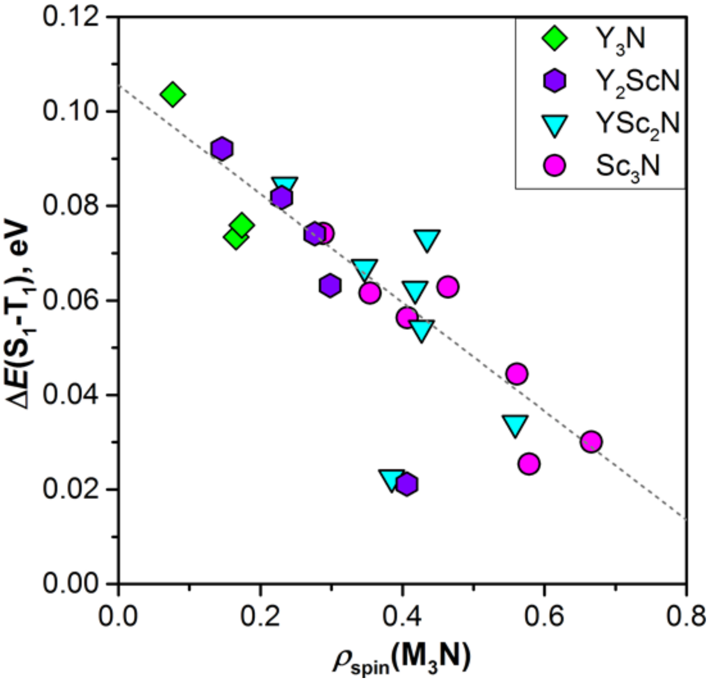

Figure S4. The relation between the total spin population of the endohedral cluster in the triplet state and the S_1–T_1 energy gap according to DFT and TD-DFT calculations. The dashed line is shown to guide the eye only.

Table S7. DFT-computed EPR parameters of selected $Y_xSc_{3-x}N@C_{80}$ conformers in the triplet state

		A_x/g_x	A_y/g_y	A_z/g_z	A_{iso}/g_{iso}	$\|e^2Qq/h\|$	η
$Y_3N@C_{80}$, **conf 1**							
PBE, ZORA	Y	1.74	1.82	2.15	1.90		
	N	−0.09	−0.09	0.82	0.21	1.359	0.00
	g	2.0024	2.0024	2.0042	2.0030		
PBE0, ZORA	Y	1.96	2.05	2.42	2.14		
	N	−0.07	−0.07	0.78	0.22	1.431	0.00
	g	2.0001	2.0001	2.0028	2.0010		
$Y_2ScN@C_{80}$, **conf 3**							
PBE, ZORA	Sc	9.10	13.65	16.61	13.12	82.5	0.06
	Y	−0.91	−1.36	−1.37	−1.22		
	Y	−5.72	−6.20	−6.31	−6.08		
	N	−0.11	−0.20	−0.35	−0.22	1.247	0.17
	g	1.9974	1.9994	2.0032	2.0000		
PBE0, ZORA	Sc	16.15	20.81	23.61	20.19	82.90	0.07
	Y	−0.97	−1.35	−1.42	−1.25		
	Y	−5.67	−6.06	−6.31	−6.01		
	N	−0.28	−0.44	−0.69	−0.47	1.311	0.18
	g	1.9978	1.9986	2.0007	1.9990		
$Y_2ScN@C_{80}$, **conf 2**							
PBE, ZORA	Sc	13.57	19.57	20.95	18.03	84.47	0.06
	Y	−1.22	−1.55	−1.67	−1.48		
	Y	−2.67	−3.01	−3.26	−2.98		
	N	−0.07	−0.20	−0.40	−0.22	1.235	0.20
	g	1.9971	2.0011	2.0024	2.0002		
PBE0, ZORA	Sc	20.53	27.03	27.86	25.14	85.05	0.07
	Y	−1.20	−1.56	−1.57	−1.45		
	Y	−3.56	−3.85	−4.17	−3.86		
	N	−0.24	−0.50	−0.77	−0.50	1.296	0.20
	g	1.9972	1.9998	2.0013	1.9994		
$YSc_2N@C_{80}$, **conf 3**							
PBE, ZORA	Sc	23.83	28.27	31.36	27.82	79.5	0.06
	Sc	19.41	25.44	26.59	23.81	81.2	0.05
	Y	−2.21	−2.64	−2.72	−2.52		
	N	−0.17	−0.31	−0.40	−0.29	1.217	0.17
	g	1.9987	2.0006	2.0016	2.0003		
PBE0, ZORA	Sc	25.88	31.03	32.64	29.85	82.0	0.05
	Sc	35.78	40.91	42.95	39.88	80.3	0.07
	Y	−2.20	−2.56	−2.62	−2.46		
	N	−0.28	−0.49	−0.75	−0.51	1.261	0.18
	g	1.9983	1.9993	2.0002	1.9993		

		A_x/g_x	A_y/g_y	A_z/g_z	A_{iso}/g_{iso}	$\|e^2Qq/h\|$	η
$Sc_3N@C_{80}$, **conf 3**							
PBE, ZORA	Sc	35.13	36.11	41.48	37.58	77.0	0.09
	N	−0.22	−0.47	−0.47	−0.39	1.316	0.00
	g	2.0002	2.0002	2.0023	2.0009		
PBE0, ZORA	Sc	44.44	46.09	49.14	46.56	78.24	0.07
	N	−0.12	−0.40	−0.40	−0.31	1.305	0.00
	g	2.0001	2.0002	2.0012	2.0005		
B3LYP, ZORA	Sc	43.89	45.00	50.52	46.47	79.08	0.08
	N	−0.11	−0.24	−0.24	−0.20	1.453	0.00
	g	1.9996	1.9996	2.0011	2.0001		

Theory: PBE or PBE0 functional with ZORA scalar-relativistic correction, the basis set is SARC or ZORA-adapted version of def2-TZVP.[7, 8, 12] Computed parameters are: Principal values of A-tensor (A_x,A_y,A_z) and their average (A_{iso}) in MHz, principal values of g-tensor (g_x, g_y, g_z) and their average (g_{iso}), nuclear quadrupolar coupling $|e^2Qq/h|$ in MHz, and asymmetry parameter η.

Comparison of experimental and computed ^{45}Sc hfc constants shows a systematic underestimation of the experimental values by theory. Similar underestimation was also observed for the ^{45}Sc hfc constants in $Sc_3N@C_{80}^-$ anion in the exhaustive study,[13] which used various density functional and basis sets. A possible reason of this underestimation is the influence of the dynamic effects on the experimental values,[14] but the failure of DFT approach to correctly describe the polarization of core s-electrons in Sc atoms is also a possibility.

Photoelectron spectroscopy

Photoelectron spectra of $Sc_3N@C_{80}^-$ were obtained using a size-selective cryogenic photoelectron spectroscopy apparatus that couples an electrospray ionization (ESI) source and a temperature-controlled ion trap to a magnetic-bottle time-of-flight photoelectron spectrometer.[15] A small sample of $Sc_3N@C_{80}$ was dissolved in toluene and reduced by adding diluted tetrakis(dimethylamino)ethylene (TDAE) in CH_3CN under a nitrogen-filled glovebox. The resulting ESI solution was ca. $5x10^{-4}$ M. $Sc_3N@C_{80}^-$ anions were directed by two rf-only quadruples into the cryogenic ion trap, where the ions were accumulated and cooled down to 12 K in order to eliminate vibrational hot bands and achieve optimal spectral resolution. The $Sc_3N@C_{80}^-$ anions were mass-selected and then maximally decelerated (to minimize Doppler Broadening) before being photodetached with 266 nm (4.661 eV) or 355 nm (3.496 eV) photons. The laser was operated at a 20 Hz repetition rate with the ion beam off on alternating laser shots for background subtraction. Photoelectrons were collected at nearly 100% efficiency by the magnetic bottle and analyzed in a 5.2 m long electron flight tube. TOF photoelectron spectra were collected and converted to kinetic energy spectra calibrated using the known spectra of I^- (ref [16]) and $OsCl_6^{2-}$ (ref [17]). The electron binding energy spectra were obtained by subtracting the kinetic energy spectra from the detachment photon energies used. The gas-phase electron affinity was directly measured from the 0–0 transition in the corresponding photoelectron spectrum.

Light-induced pulsed EPR

Saturated solutions of $Y_3N@C_{80}$, $Y_2ScN@C_{80}$, $YSc_2N@C_{80}$ and $Sc_3N@C_{80}$ were prepared in toluene-d_8 (Sigma-Aldrich) at ambient conditions (without degassing), filled in the W-band quartz tubes, and flash-frozen by immersing into the liquid nitrogen before insertion into the microwave cavity of the spectrometer.

Sample illumination in EPR/ENDOR experiments

The continuous irradiation in W-band EPR/ENDOR experiments was achieved with the Power Technology Inc. IQ1C laser (510 nm, 40mW). A pulsed Nd-YAG Innolas SpitLight Compact 400 laser equipped with OPO (1.5 mJ/pulse for W-band, 3 mJ/pulse for X-band at 488 nm) was used in time-resolved measurements.

W-band pulsed EPR measurements

W-band measurements were performed at 20 K using a Bruker ELEXSYS E680 spectrometer operating at about 94 GHz. All experiments were carried out with a homebuilt ENDOR microwave cavity.[18-20] Electron spin echo-detected (ESE) field-swept spectra were measured using the Hahn echo pulse sequence t_p−τ−2t_p−τ−echo with t_p = 20 ns and τ = 300 ns. In continuous irradiation experiments (510 nm, 40 mW), 20-100 echoes were accumulated depending on the S/N and integrated over 140 ns around their maximum at each field position. The pulse repetition time was set to 2 ms. In the time-resolved EPR measurements, 12 ns laser pulse and a 500 ns delay after laser flash (t_{DAF}) preceded the Hahn echo sequence. Four echoes per field point were recorded with a repetition time of 2.5 s (laser repetition time). The decay of the polarized ESE signals was followed by incrementing the t_{DAF} in the range of 0.5µs-900 ms.

The Mims-type ENDOR spectra were measured at 20 K under continuous irradiation of the sample (510 nm, 40 mW) using the Mims-type ENDOR sequence t_p−τ−t_p−t_{RF}−t_p−τ−echo, with an RF pulse applied during the time interval t_{RF}. The experimental conditions were t_p = 40 ns, t_{RF} = 48 µs or 60 µs, and τ = 564 ns. All ENDOR spectra were recorded using the stochastic acquisition mode with two shots for each point, and the total number of scans was varied in the range 100−400 depending on the S/N.

The Davies-type ENDOR spectra were measured at 20 K under continuous irradiation of the sample (510 nm, 40 mW) using the ENDOR sequence t_{inv}−t_{RF}−t_p−τ−2t_p−τ−echo. The experimental conditions were t_{inv} = 100 ns, t_p = 40 ns, t_{RF} = 10 µs and τ = 1000 ns. Davies-type ENDOR spectra were recorded using the stochastic acquisition mode with two shots for each point, and the total number of scans was varied in the range 500−1000 depending on the S/N.

The EPR and ENDOR spectra were analyzed by computer simulation within the Spin Hamiltonian framework sketched in the manuscript using the EasySpin[21] package running under Matlab.

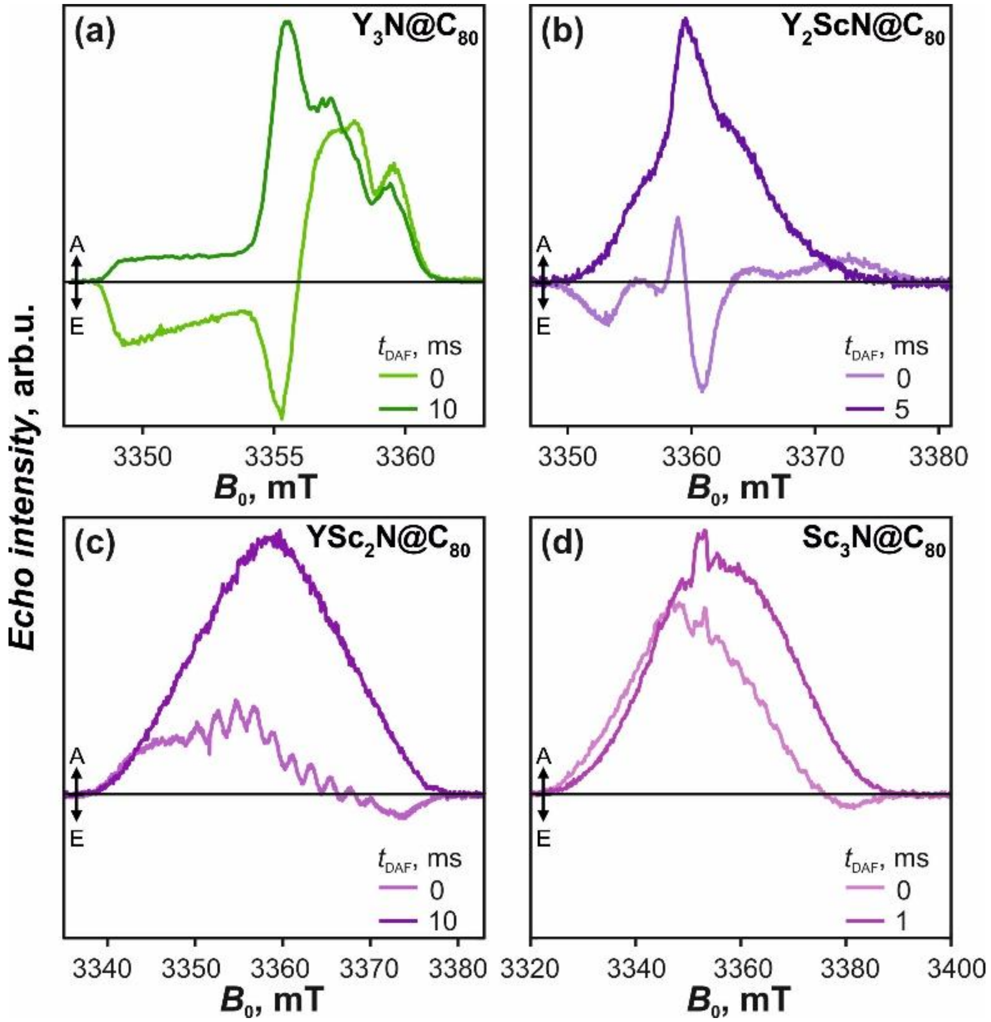

Figure S5. W-band (94 GHz) time-resolved ESE EPR spectra of the EMF triplets from a) $Y_3N@C_{80}$, b) $Y_2ScN@C_{80}$, d) $YSc_2N@C_{80}$ and d) $Y_3N@C_{80}$. The pale lines show the polarized spectra recorded directly after the laser flash while the full colors represent records after t_{DAF} of few ms when the polarization already relaxed. The polarized spectra (pale lines) reflect the non-equilibrium population of triplet energy levels built during the intersystem crossing and include absorptive (A) and emissive (E) signals. The "relaxed" spectra (full colors) correspond to the thermal Boltzmann population of the triplet levels and are purely absorptive.

References

1. Chen, N.; Fan, L. Z.; Tan, K.; Wu, Y. Q.; Shu, C. Y.; Lu, X.; Wang, C. R., Comparative Spectroscopic and Reactivity Studies of $Sc_{3-x}Y_xN@C_{80}$ (x = 0-3). *J. Phys. Chem. C* **2007,** *111* (32), 11823-11828.
2. Zhang, Y.; Krylov, D.; Rosenkranz, M.; Schiemenz, S.; Popov, A. A., Magnetic Anisotropy of Endohedral Lanthanide Ions: Paramagnetic NMR Study of $MSc_2N@C_{80}$-I_h with M running through the Whole 4f Row. *Chem. Sci.* **2015,** *6*, 2328-2341.
3. Laikov, D. N.; Ustynuk, Y. A., PRIRODA-04: a quantum-chemical program suite. New possibilities in the study of molecular systems with the application of parallel computing. *Russ. Chem. Bull.* **2005,** *54* (3), 820-826.
4. Laikov, D. N., Fast evaluation of density functional exchange-correlation terms using the expansion of the electron density in auxiliary basis sets. *Chem. Phys. Lett.* **1997,** *281*, 151-156.
5. Neese, F., Software update: the ORCA program system, version 4.0. *WIREs Comput. Mol. Sci.* **2018,** *8* (1), e1327.
6. Neese, F., The ORCA program system. *WIREs Comput. Mol. Sci.* **2012,** *2* (1), 73-78.
7. Pantazis, D. A.; Chen, X.-Y.; Landis, C. R.; Neese, F., All-Electron Scalar Relativistic Basis Sets for Third-Row Transition Metal Atoms. *J. Chem. Theory Comput.* **2008,** *4* (6), 908-919.
8. Weigend, F.; Ahlrichs, R., Balanced basis sets of split valence, triple zeta valence and quadruple zeta valence quality for H to Rn: Design and assessment of accuracy. *Phys. Chem. Chem. Phys.* **2005,** *7*, 3297-3305.
9. Neese, F., Efficient and Accurate Approximations to the Molecular Spin-Orbit Coupling Operator and their use in Molecular g-Tensor Calculations. *J. Chem. Phys.* **2005,** *122*, 034107.
10. Neese, F., Prediction of Electron Paramagnetic Resonance g-values by Coupled Perturbed Hartree-Fock and Kohn-Sham Theory. *J. Chem. Phys.* **2001,** *115*, 11080-11096.
11. Neese, F.; Wennmohs, F.; Becker, U.; Riplinger, C., The ORCA quantum chemistry program package. *J. Chem. Phys.* **2020,** *152* (22), 224108.
12. Pantazis, D. A.; Neese, F., All-Electron Scalar Relativistic Basis Sets for the Lanthanides. *J. Chem. Theory Comput.* **2009,** *5* (9), 2229-2238.
13. Popov, A. A.; Dunsch, L., Hindered Cluster Rotation and ^{45}Sc Hyperfine Splitting Constant in Distonoid Anion Radical $Sc_3N@C_{80}$, and Spatial Spin Charge Separation as a General Principle for Anions of Endohedral Fullerenes with Metal-Localized Lowest Unoccupied Molecular Orbitals. *J. Am. Chem. Soc.* **2008,** *130* (52), 17726-17742.
14. Popov, A. A.; Dunsch, L., Charge controlled changes in the cluster and spin dynamics of $Sc_3N@C_{80}(CF_3)_2$: the flexible spin density distribution and its impact on ESR spectra. *Phys. Chem. Chem. Phys.* **2011,** *13* (19), 8977-8984.
15. Wang, X.-B.; Wang, L.-S., Development of a low-temperature photoelectron spectroscopy instrument using an electrospray ion source and a cryogenically controlled ion trap. *Rev. Sci. Instrum.* **2008,** *79* (7), 073108.
16. Hanstorp, D.; Gustafsson, M., Determination of the electron affinity of iodine. *J. Phys. B: At. Mol. Opt. Phys* **1992,** *25* (8), 1773-1783.
17. Wang, X.-B.; Wang, L.-S., Photodetachment of free hexahalogenometallate doubly charged anions in the gas phase: $[ML_6]^{2-}$, (M=Re, Os, Ir, Pt; L=Cl and Br). *J. Chem. Phys.* **1999,** *111* (10), 4497-4509.
18. Cox, N.; Lubitz, W.; Savitsky, A., W-band ELDOR-detected NMR (EDNMR) spectroscopy as a versatile technique for the characterisation of transition metal–ligand interactions. *Molecular Physics* **2013,** *111* (18-19), 2788-2808.

19. Nalepa, A.; Möbius, K.; Lubitz, W.; Savitsky, A., High-field ELDOR-detected NMR study of a nitroxide radical in disordered solids: Towards characterization of heterogeneity of microenvironments in spin-labeled systems. *Journal of Magnetic Resonance* **2014,** *242*, 203-213.
20. Rapatskiy, L.; Cox, N.; Savitsky, A.; Ames, W. M.; Sander, J.; Nowaczyk, M. M.; Rögner, M.; Boussac, A.; Neese, F.; Messinger, J.; Lubitz, W., Detection of the Water-Binding Sites of the Oxygen-Evolving Complex of Photosystem II Using W-Band 17O Electron–Electron Double Resonance-Detected NMR Spectroscopy. *Journal of the American Chemical Society* **2012,** *134* (40), 16619-16634.
21. Stoll, S.; Schweiger, A., EasySpin, a comprehensive software package for spectral simulation and analysis in EPR. *J. Magn. Reson.* **2006,** *178* (1), 42-55.
